# Physical activity–based, wholistic, wellness interventions for Indigenous women in Canada: An environmental scan to identify programs and promising practices

**DOI:** 10.17269/s41997-025-01091-9

**Published:** 2025-11-06

**Authors:** Sonja Wicklum, Tia Black, Loretta Tuttauk, Lynden Lindsay Crowshoe, Yunqi Jacob Ji, Kerry McBrien, Jessica Zhang, Carly Checholik, Ashley Amson, Patricia Doyle-Baker, Alicia Oliver, Levi Frehlich

**Affiliations:** 1 Department of Family Medicine, Cumming School of Medicine, University of Calgary, Health Sciences Centre (G329), 3330 Hospital Drive NW, Calgary, AB Canada; 2 North East Family Connections, 95 Falshire Drive NE, Calgary, AB Canada; 3 Miskanawah, 2716 Sunridge Way NE, Calgary, AB Canada; 4 Department of Community Health Sciences, Cumming School of Medicine, University of Calgary, 3280 Hospital Drive NW, Calgary, AB Canada; 5 Department of Mathematics and Statistics, Mathematical Sciences 476, University of Calgary, 2500 University Drive NW, Calgary, AB Canada; 6 Mosaic Primary Care Network, 807 Manning Rd NE, Calgary, AB Canada; 7 Faculty of Medicine and Dentistry, University of Alberta, 2J2.00 Walter J MacKenzie, Health Sciences Centre, Edmonton, AB Canada; 8 Interdisciplinary School of Health Sciences, University of Ottawa, Thompson Hall – THN 136 25 University Private, Ottawa, ON Canada; 9 Faculty of Kinesiology, University of Calgary, 2500 University Drive NW, Calgary, AB Canada; 10 Onion Lake Health Board, PO Box 70, Onion Lake, SK Canada

**Keywords:** Indigenous, Women, Prevention, Physical activity, Community programming, Intervention, Health programming, Autochtone, Femmes, Prévention, Activité physique, Programmes communautaires, Intervention, Programmes de santé

## Abstract

**Objectives:**

This environmental scan has two aims. The first is to identify wholistic, physical activity (PA)–based wellness interventions for Indigenous women in Canada through the completion of a scoping review of published and grey literature and key informant interviews. The second is to identify promising practices and potential barriers to intervention development and delivery.

**Methods:**

Components of the environmental scan included (1) the creation of a logic model in collaboration with a community-based Advisory Group, (2) a scoping review of wholistic PA-based wellness interventions between January 1990 and March 2022, (3) key informant interviews of individuals involved in PA-based wellness programming, and (4) thematic analysis of promising practices, enablers, and barriers found in both the articles and interviews.

**Synthesis:**

The scoping review identified 16 interventions. Through key informant interviews, 6 additional interventions were identified. Programs were largely community-based and culturally appropriate. Financial factors were the most common barrier. Walking as a form of PA was commonly employed and key informant interviews highlighted the importance of exploring on-the-land activities, group activities, and the incorporation of all elements of wholistic health (mental, physical, spiritual, and emotional). Only one study employed Indigenous research methods.

**Conclusion:**

There is limited published literature and a dearth of PA-based, wholistic health programs for Indigenous women in Canada. More programming and research are required to address the unique health needs of Indigenous women and mitigate the legacy impacts of colonization on health.

**Supplementary Information:**

The online version contains supplementary material available at https://doi.org/10.17269/s41997-025-01091-9.

## Résumé

**Objectifs:**

Notre analyse environnementale avait deux buts. Le premier était de repérer les interventions de mieux-être holistiques fondées sur l’activité physique (AP) à l’intention des femmes autochtones au Canada en menant une étude de champ des articles publiés et de la littérature grise et en organisant des entretiens avec des informateurs et des informatrices. Le second était de repérer les pratiques prometteuses et les obstacles possibles à l’élaboration et à la prestation des interventions.

**Méthodes:**

Les éléments de l’analyse environnementale étaient 1) la mise au point d’un modèle logique en collaboration avec un groupe consultatif communautaire, 2) une étude de champ des interventions de mieux-être holistiques fondées sur l’AP menées entre janvier 1990 et mars 2022, 3) des entretiens avec des personnes impliquées dans la programmation d’interventions de mieux-être fondées sur l’AP et 4) l’analyse thématique des pratiques prometteuses, des incitateurs et des obstacles relevés dans la littérature et dans les entretiens.

**Synthèse:**

L’étude de champ a identifié 16 interventions. Six autres ont été identifiées lors des entretiens avec des informateurs et des informatrices. Les programmes étaient dans une large mesure communautaires et culturellement appropriés. Les facteurs financiers représentaient l’obstacle le plus commun. La marche était couramment employée comme forme d’AP, et les entretiens ont souligné l’importance d’explorer les activités sur le territoire, les activités de groupe et l’intégration de tous les aspects (mental, physique, spirituel et émotionnel) de la santé holistique. Une seule étude faisait appel à des méthodes de recherche autochtones.

**Conclusion:**

Il y a peu d’articles publiés et un manque criant de programmes de santé holistiques fondés sur l’AP à l’intention des femmes autochtones au Canada. Il faut davantage de programmes et d’études pour aborder les besoins de santé particuliers des femmes autochtones et atténuer les séquelles de la colonisation sur la santé.

## Introduction

Indigenous Peoples in Canada experience greater ill health across multiple domains. This is evidenced by a higher prevalence of obesity, mental health disorders, and other associated diseases, including cardiovascular disease, diabetes, autoimmune diseases, arthritis, cancer, substance use disorders, and suicide (Bombay et al., 2014; Government of Canada, 2011; Walker et al., 2020; Young et al., 2000). Reasons for these disparities in Canada are multifactorial and include social and material consequences of colonialism including structural inequities in health, education, social services and other systems, chronic and overwhelming stress from social (e.g., discrimination) and systemic exclusion, anti-Indigenous racism (Turpel-Lafond, 2021), poor nutrition, decreased physical activity (PA), and genetic (e.g., specific polymorphism identified in Manitoba Oji-Cree) and epigenetic predisposition (Atlantic Centre of Excellence in Women’s Health, 2009; Henderson et al., 2020). Indigenous women, in particular, face disproportionately poor health outcomes when compared to both Indigenous men and non-Indigenous Canadians. For example, a recent study of Ontario First Nations revealed that diabetes is more prevalent in First Nations women, and at younger ages, compared to non-First Nations (Walker et al., 2020). Another reason cited for this higher prevalence relates to the added burden of patriarchal power relations and sexism (Native Women’s Association of Canada, 2007).

Regular PA serves as a preventive measure against obesity and other chronic diseases, offering numerous health benefits. Unfortunately, evidence indicates that PA levels drop significantly during adolescence (Dumith et al., 2011; Sember et al., 2020). Further, PA rates are lower for female gender youth, and fewer adult women participate in sport (Colley et al., 2011; Dumith et al., 2011; Johnstone & Millar, 2012). This is even more pronounced for Indigenous women (Young et al., 2000). As identified in the *Truth and Reconciliation Commission Report of Canada*, decreased rates of participation in PA among Indigenous women are due to both structural and sociocultural barriers (Atlantic Centre of Excellence in Women’s Health, 2009; Truth & Reconciliation Commission of Canada, 2015). Ferguson and Philipenko (2015) found female Indigenous university students had unique needs, longed for tradition, and have an extended personal connection with PA, which was challenging to access (Ferguson & Philipenko, 2015). Indigenous women’s perspectives on the causes of PA decrease are generally not well studied or studied through the eyes of white researchers (Ratna & Samie, 2017).

PA, through an Indigenous lens, can be viewed as more than a way to simply improve cardiovascular fitness, but rather as an integral part of Indigenous culture and of wholistic health (Hoffman, 2013). Wholistic health is an interconnected and balanced approach that considers the harmony and well-being of all aspects of life, including the spiritual, physical, mental, and environmental dimensions, within the context of community and nature (Miles et al., 2023). As such, scholars have begun to explore PA as a way to heal and to foster decolonization (McGuire-Adams, 2020; McGuire-Adams & Giles, 2018). Of note, we use the spelling ‘wholistic’ throughout this paper in concordance with the Indigenous view of health (Miles et al., 2023).

Community programming is one means of increasing PA as well as providing nutritional knowledge—two factors known to reduce the risk of obesity-related illness. However, these programs are limited in most Indigenous communities. Previous programming has highlighted the importance of culturally relevant interventions that acknowledge the significance of Indigenous practices and customs, and the need for collaborative efforts between Indigenous communities and policymakers to create initiatives that support and celebrate Indigenous women’s PA, fostering wholistic health and wellness (Skinner et al., 2012). Indigenous Nations, communities, and cultures possess a wealth of knowledge, skills, and structures for healthful living, and Indigenous women are integral to each of these.

PA programming for Indigenous Peoples is growing in Canada. However, it is often focused on male adult sports and PA in children. In 2017, Pelletier completed a systematic review of PA interventions for Indigenous adults in Canada (Pelletier et al., 2017), highlighting the lack of interventions and robust evaluations, validating the need for culturally relevant educational and measurement tools. Most concerning is a perceived lack of programming available to Indigenous women. A systematic review of PA-based interventions specific to Indigenous women internationally was completed by Wicklum et al. (2019, 2021). It found marked variability in programming and study design and as a result, it was challenging to draw conclusions about effectiveness and best practices (Wicklum et al., 2021). The perceived dearth in programming, combined with the fact that many programs may not formally evaluate or publish results, established the need for an environmental scan.

## Methods

### Overview

This environmental scan will (1) determine if this perceived lack of programming is valid, (2) review existing programs, and (3) assist organizations attempting to offer preventative health interventions for Indigenous women throughout Canada, by identifying ‘promising practices’ that can inform existing and future interventions. A strategic focus of this study was to improve an existing intervention, Makoyoh’sokoi (the Wolf Trail Program), a 15-week wholistic, wellness program for Indigenous women that has been running in urban and rural centres in Alberta and Saskatchewan, two provinces in the Canadian prairies (Table 1) (Frehlich et al., 2023). This community-based programming is inclusive of all women, including cis-gender, transgender, non-binary, and two-spirited individuals and as such, when referring to women throughout this paper, it is reflective of all women. The protocol is available at https://doi.org/10.17605/OSF.IO/A54X9. The objective is to then apply these ‘promising practices’ to the further development of Makoyoh’sokoi—The Wolf Trail Program, www.wolftrail.ca.

**Table 1:** Makoyoh’sokoi (The Wolf Trail Program) description (www.wolftrail.ca)

15-week^a^ duration
Physical activity–based, wholistic wellness program
Weekly physical activity (wide variety, Indigenous options e.g. Pow Wow dance), nutrition education, and cultural elements
Cultural elements: Guidance by Elders and an Advisory Group Ceremony—Spring ceremony of entire Makoyoh’sokoi project to honor naming and individual group ceremony Elder teaching during program Weekly sharing circle Medicine Wheel teachings and exercises applied
Roles and origin: (1)Program Facilitators from community (2)Facilitator Lead from community (3)Community Leads from community—support program Facilitators, typically hold positions as health or recreation directors
Participating communities with leadership involved in this study^b^: Edmonton (AB) Calgary (AB) Onion Lake Cree Nation (AB/SK) Waterhen Lake First Nation (SK) Flying Dust First Nation (SK) Ministikwan Lake First Nation (SK)
Participating community organizations: Miskanawah (Calgary) City of Calgary North East Family Connections (Calgary, AB) Indigenous Congress of Alberta Association (Edmonton, AB) Onion Lake Health Centre (Onion Lake, AB) Meadow Lake Tribal Council (Meadow Lake, SK)
Protocol paper available (Zhang et al., 2023)

^a^New program length as of Sept 2023

^b^At time of this study, April 2022

Environmental scans can be a valuable tool to define and inform areas of health research for which there are limited publications but immense practical knowledge because of how different types of knowledge are captured in the process. They can support developing the ‘big picture’. Environmental scans were originally developed in the area of business landscape as a tool to organize data and inform decision-making (Reichel & Preble, 1984); they have been used in many areas of health research: public health, medical education, and community-based research (Turin et al., 2020). Environmental scans are a Western methodology; however, they have been used when studying Indigenous populations. Examples include the assessment of mental health programming (ACCESS Open Minds, n.d.), sport and recreation programming (Sutherland, 2021), and health data use (Wright 2022).

This study adapts the environmental scan methodology as outlined by Shahid and Turin (Shahid & Turin, 2018) and was chosen because it specifically emphasizes an iterative process and inclusion of community knowledge and perspectives. Our fundamental aim was to identify the ‘promising practices’ that could inform the Makoyoh’sokoi program. This aim was agreed upon by the Advisory Group and Community Leads for the Makoyoh’sokoi program. The former is composed of Elders, community leaders, and a past Facilitator. The latter are the group of individuals that have advocated for and enabled the Makoyoh’sokoi program to be developed in their community. Like the Advisory Group, they are individuals based in community with intimate knowledge of the health challenges faced by local Indigenous women.

**Fig. 1:**
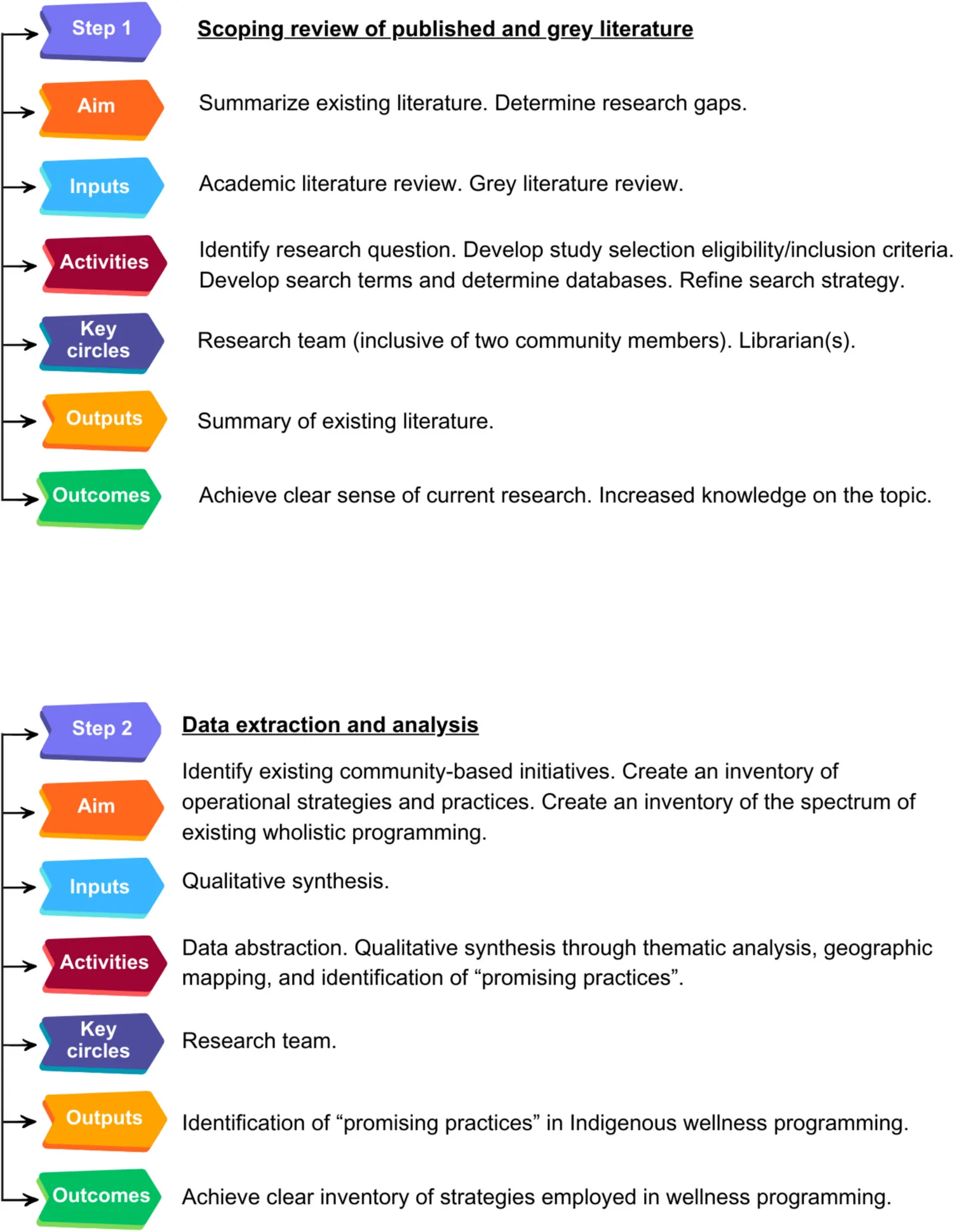

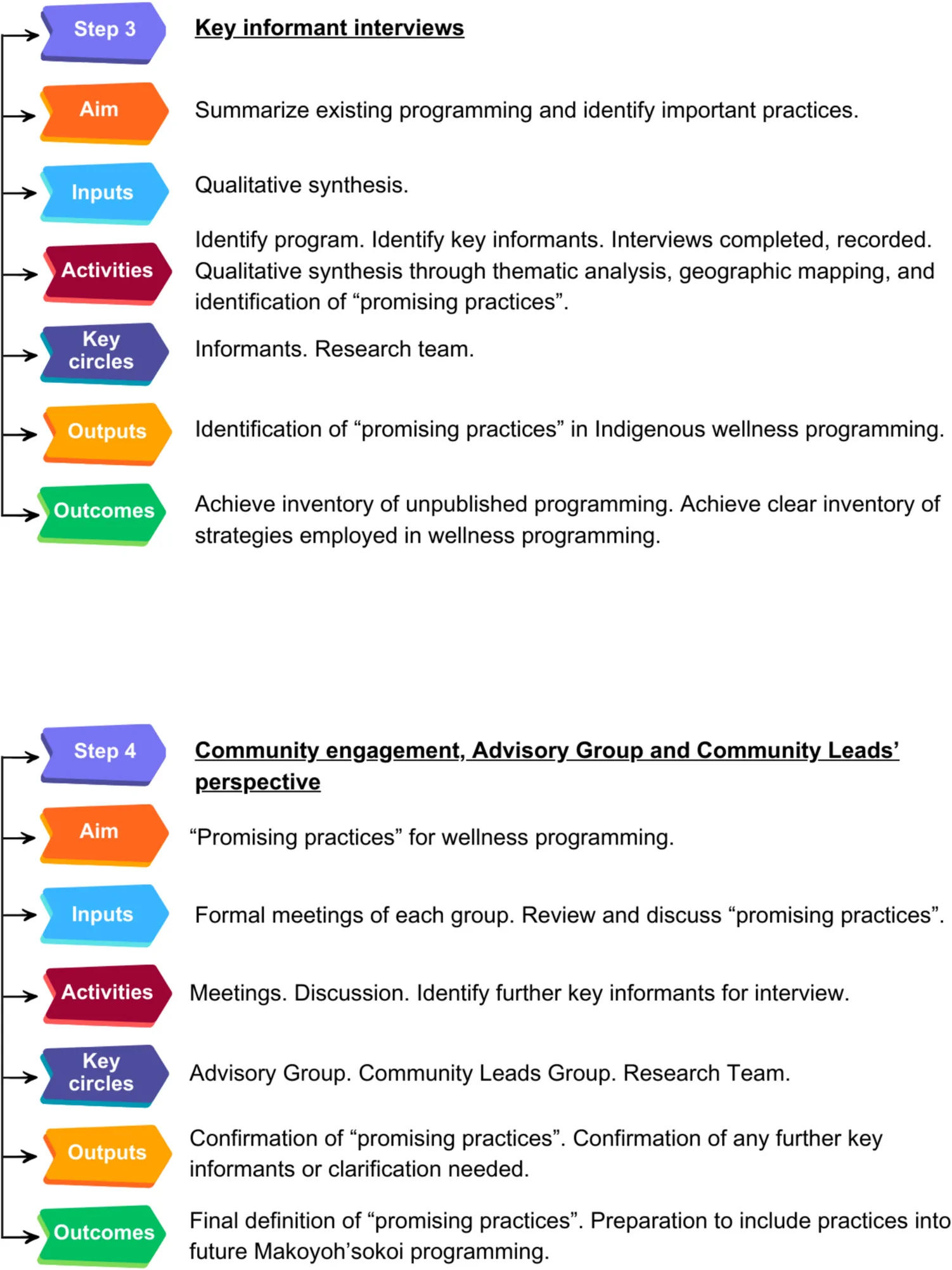
Logic map for the environmental scan of physical activity–based wellness program for Indigenous women in Canada

We first created a logic map informed by Turin et al.’s study of community-based health and wellness literacy initiatives (Turin et al., 2020) (Fig. 1). The outcome of ‘Preparation to include practices into future Makoyoh’sokoi programming’ is not reported in this article as it is beyond the scope of this review. However, it is included in our logic map to illustrate the practical endpoint of this study.

### Scoping review

The scoping review follows the Preferred Reporting Items for Systematic Reviews and Meta-analyses Protocols – extension for Scoping Reviews (PRISMA-ScR) (Tricco et al., 2018). The purpose of the scoping review was to identify published PA-based, wholistic wellness interventions for Indigenous adult women in Canada and identify ‘promising practices’. For our purposes, ‘wholistic’, as an extension to solely PA-based wellness, is meant to include programming that extends to other aspects of wholistic health, defined as spiritual, emotional, and mental, as discussed through oral tradition in Indigenous populations, often referred to as the components of the Medicine Wheel (Miles et al., 2023). It is very important to note that there are many types of Medicine Wheels and views on wholistic health among Indigenous populations and the Medicine Wheel is not a paradigm that is used by all First Nations communities.

We completed a preliminary search on PubMed and identified a 2017 review article (Pelletier) that focused on PA interventions to improve health for Indigenous adults. The authors kindly shared their search terms. In consultation with our research team, Community Leads, and a medical librarian, we recognized the need to expand our search with the following additions: (1) terms to thoroughly capture indigeneity, (2) terms to include traditional physical activities (such as ‘on-the-land’ activities, fishing, hunting), and (3) the addition of three databases (Scopus, Web of Science, and Canadian Business and Current Affairs) to their original four (Medline, PsycINFO, EMBASE, CINAHL). The multi-search strategy is outlined in Table 2. In total, we searched eight databases (OVID Medline, PubMed, CINAHL Complete, PsycINFO, OVID EMBASE, Scopus, Web of Science, Canadian Business and Current Affairs). Further, the first 20 pages of Google Scholar were searched before reaching clear lack of relevance. Due to lack of gender-specific programming, we widened our search to include all adults.

**Table 2:** Overview of multi-search strategy for scoping review

Searches conducted	Overview of search
A: Repeated Pelletier’s search (Pelletier et al., 2017)	Used stated search terms and stated databases
B: Difference search (1990–Feb 2017) = Comprehensive Search (A) minus Pelletier Search (ref)	Both traditional physical activity and additional Indigenous terms were added to Pelletier’s original search to complete a comprehensive search for this time period. Then, to discover studies picked up by our supplemented search terms only, we subtracted Pelletier’s
C: Additional databases search (1990–Feb 2017)	Comprehensive search terms applied to the three additional databases not included in the Pelletier search (Scopus, Web of Science, and Canadian Business and Current Affairs)
D: Updated search (Feb 2017–Mar 2022)	Updated search of entire supplemented search terms across all databases

We limited our search to (1) Canadian programs; (2) peer-reviewed journal articles, dissertations/theses, and grey literature; (3) programs for Indigenous (First Nation, Inuit, and Métis) adults 18 years and older; and (4) programs that included a PA-based interventions. We excluded organized sports and programs that did not intentionally address at least one other component of wholistic health—spiritual, mental, or emotional. The search strategies for databases can be found in Supplementary Table A. Further, we searched the websites of known organizations that provide PA-based programming to determine if there were program reports available, not otherwise found in our database search.

Article tracking, review, and extraction were completed on Covidence (Covidence systematic review software, Veritas Health Innovation, Melbourne, Australia. Available at covidence.org). Duplicates were removed. Screening and full article review were completed by two or more independent reviewers (JZ, CC, LF, SW) with all disagreements concluded by a third reviewer after discussion.

Data extracted from individual articles included title, author, year, location, program goals and description, specifically looking for presence or absence of spiritual (cultural), emotional, or mental (including nutrition education) elements, evaluation summary if available, identified barriers and enablers, and limitations of the study.

### Key informant interviews

Community Leads and the Advisory Group were involved in interview design. Key informants were identified by Community Leads, contacting programs identified in the literature, online searches, and word-of-mouth from previously identified knowledge experts. Specifically, Indigenous sport and wellness circles and friendship centres were identified as important contacts. The websites of 18 sport and wellness circles across Canada were searched; 13 relevant programs were contacted, resulting in seven interviews. The websites of 34 Indigenous friendship centres were searched to assess for relevant programs; six programs were contacted, one interview resulted.

A total of 15 key informants were identified for interviews: six from organizations with PA-based programming specific to Indigenous women, three from organizations with programming, non-PA-based, but specific to Indigenous women, and seven from organizations with general expertise in PA and Indigenous populations. Interviews (*n* = 15) were completed from March to June 2021. Makoyoh’sokoi program interview was excluded to mitigate bias as the authors have participated in the design and evaluation of the program; however, basic program information is presented in Supplementary Table B.

Interviewees were asked a series of program-specific questions and overarching environmental questions, as applicable. They included the (1) intervention description and aim with specific questions on incorporation of cultural elements and community involvement, (2) enablers and barriers, (3) evaluation strategies, (4) marketing methods, funding, and strategic partners, and where applicable (5) impact of the COVID-19 pandemic. The pandemic began in early 2020 and forced the temporary cancellation of in-person, group programming throughout Canada. Answers to these questions were tabulated by two researchers (SW, JZ). Interviewees were not compensated for participation.

Ethics for the interview component of the study was obtained through the University of Calgary Conjoint Research Ethics Board (REB 20–1002). Verbal informed consent was obtained from all interviewees at the start of the interview. All interviews were completed virtually, in English, and lasted 30–60 min. Each interview was completed by two researchers: one lead and one supporting (SW, PE, JZ). Thematic analysis was completed by two researchers (SW, PE, JZ), with discrepancies resolved by a third (JZ, CC). The Advisory Group recommended anonymity of interviewees be maintained throughout the process; identifiers have been removed from quotes and tables. An anonymized summary of interview answers to questions is provided in Supplementary Table B.

The tabulated analysis of both the articles identified in the scoping review and interview question responses was shared with the Makoyoh’sokoi Advisory Group, Community Leads, and the research team, including two community members, to determine common themes.

## Synthesis

### Scoping review

**Fig. 2:**
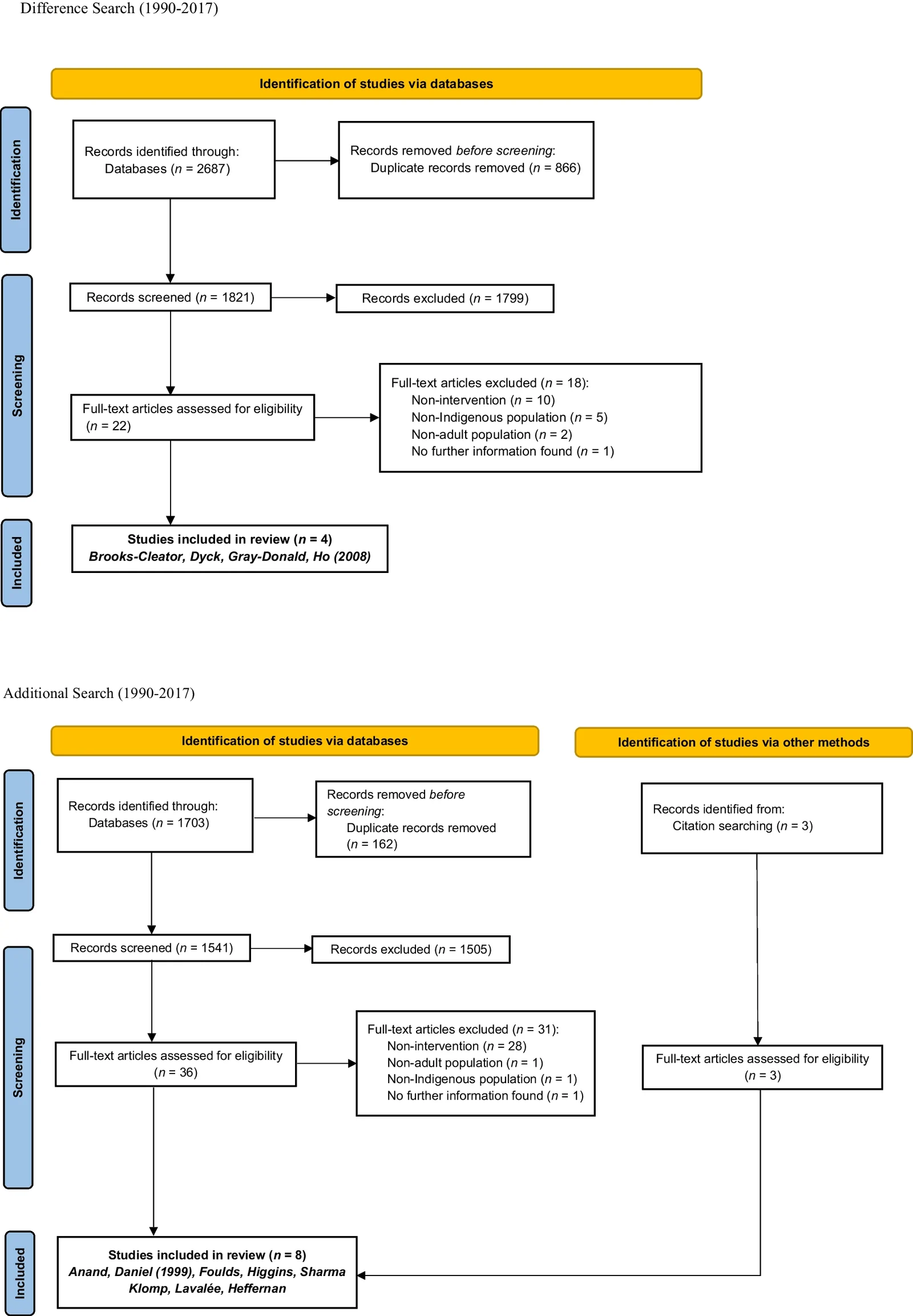

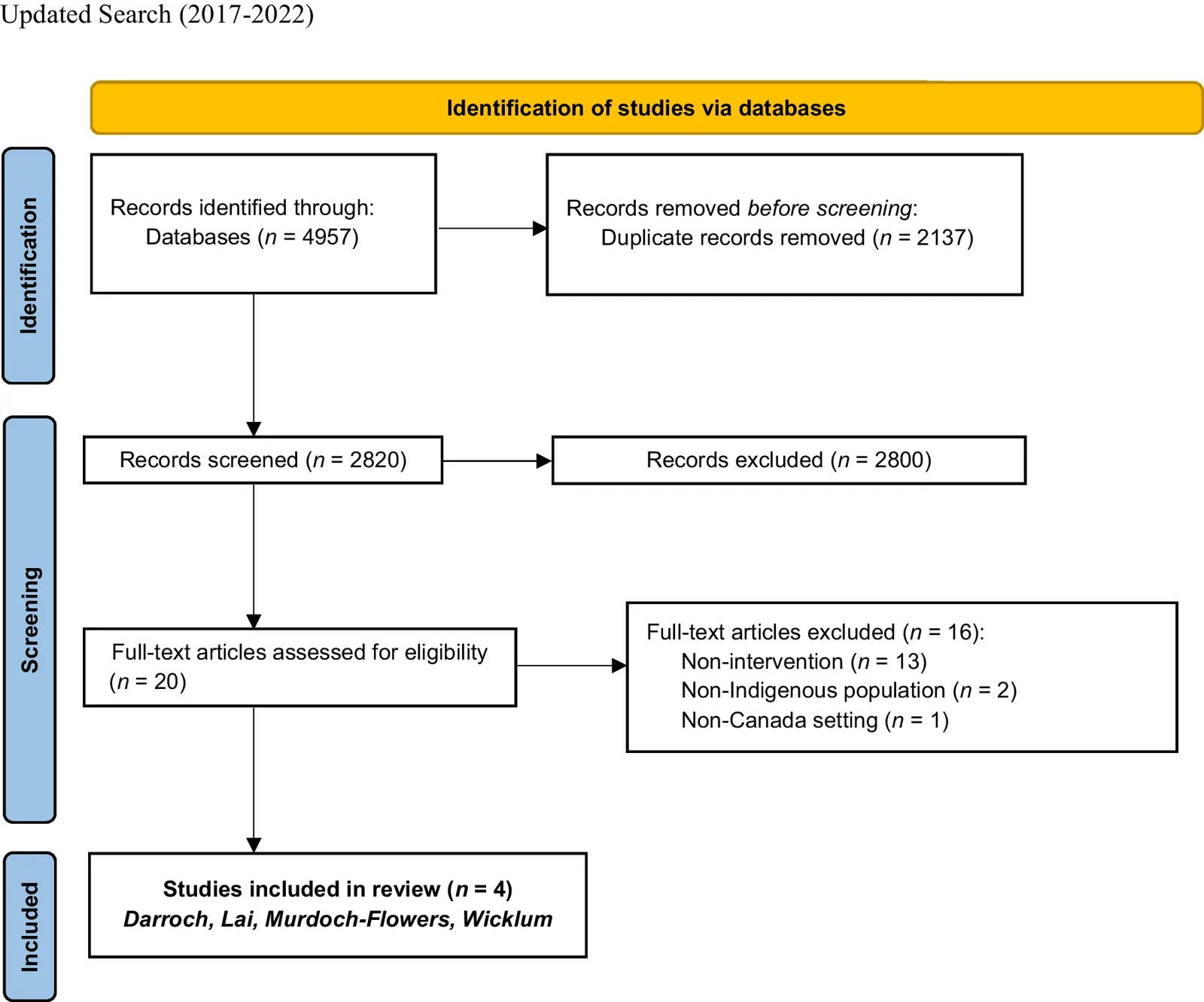
PRISMA flow diagrams for each search

The PRISMA flow diagram for searches described in Table 2 are presented in Fig. 2. A summary of the PRISMA flowchart is found in Table 3. Results were cross-checked to assess for inconsistencies. In summary, Pelletier’s search identified four articles for review and our updated database and ‘other sources’ searches led to a further 12 articles for review, for a total of 16 articles.

**Table 3:** Summary of PRISMA flowchart for the three new primary searches utilized

Search	Database records	Citation searching records	Duplicate records removed	Screened records	Excluded records	Eligible assessed full text	Excluded full text	Total included studies
B: Difference search (1990–Feb 2017) = Comprehensive minus Pelletier (Pelletier et al., 2017)^b^	2687		866	1821	1799	22	18	4
C: Additional databases search (1990–Feb 2017)	1703	2	162	1541	1505	38	31	7^a^
D: Updated search (Feb 2017–Mar 15 2022)	4957		2137	2820	2800	20	16	4
**Total**	**9347**	**2**	**3165**	**6182**	**6104**	**78**	**65**	**15**

^a^Two articles identified from references in articles and added as from ‘other sources’

^b^Pelletier identified 5 articles to include in their narrative synthesis. Our Search B, ‘Difference’ search, identified two of the articles again, likely because Pelletier identified seven articles through ‘outside sources’ which our search identified through our expanded search terms. Similarly, our Search C, ‘Additional Databases Search’, identified another two articles, likely among those articles found by Pelletier through ‘outside sources’

Data extraction from the articles is summarized in Table 4. A total of 17 articles representing 16 interventions, from 1998 to 2022 were assessed, with the majority from the larger Canadian provinces, British Columbia, and Ontario. Intervention types varied widely, with seven focused on increasing PA and improving lifestyle (Anand et al., 2007; Brooks-Cleator & Giles, 2016; Foulds et al., 2011; Kolahdooz et al., 2014; Lai et al., 2019; Lavallée, 2007; Sharma et al., 2010; Wicklum et al., 2019), five were diabetes prevention programs (Daniel et al., 1999; Dyck et al., 1998; Heffernan et al., 1999; Higgins et al., 2004; Ho et al., 2008; Murdoch-Flowers et al., 2019), and three were PA programs for pregnant women (Darroch & Giles, 2017; Gray-Donald et al., 2000; Klomp et al., 2003). Most had small sample sizes except for Foulds et al.’s Hearts in Training program (Foulds et al., 2011). All but one involved elements of community consultation and those authors highlighted that not tailoring to the community had a negative influence on this program. In one circumstance, the development of the program was the intervention (Darroch & Giles, 2017). Many programs had community members involved in the delivery of the program and highlighted this as important and contributing both to the cultural relevancy and safety of the programming, as community members were aware of the systemic issues faced by the local participants (Anand et al., 2007; Daniel et al., 1999; Dyck et al., 1998; Gray-Donald et al., 2000; Higgins et al., 2004; Ho et al., 2008; Klomp et al., 2003; Kolahdooz et al., 2014; Lai et al., 2019; Lavallée, 2007; Murdoch-Flowers et al., 2019; Sharma et al., 2010; Wicklum et al., 2019). Three programs clearly indicated that all four quadrants of the Medicine Wheel were addressed (Lavallée, 2007; Murdoch-Flowers et al., 2019; Wicklum et al., 2019). Some programs reported positive findings beyond PA changes including significant increase in nutrition knowledge, healthy food acquisition, and healthier food preparation (Ho et al., 2008; Kolahdooz et al., 2014; Sharma et al., 2010); changes in confidence participating in group exercise and achieving PA and nutrition goals (Wicklum et al., 2019); reductions in sedentary behaviours (Anand et al., 2007); learning mind-focusing and relaxation techniques (Murdoch-Flowers et al., 2019); improvement in anthropometric measures and serum biochemistries (Heffernan et al., 1999); self-reported improvement of all four aspects of Medicine Wheel (Lavallée, 2007); and strength of commitment to diabetes prevention (Daniel et al., 1999). Of the seven programs that attempted to measure improvements in PA (Anand et al., 2007; Daniel et al., 1999; Foulds et al., 2011; Gray-Donald et al., 2000; Ho et al., 2008; Lai et al., 2019; Wicklum et al., 2019), three had positive results (Foulds et al., 2011; Lai et al., 2019; Wicklum et al., 2019), improvement in self-reported PA scores pre/post Hearts in Training learn to walk/run program (Foulds et al., 2011), improvements in self-reported weekly total step counts through the course of an 8-week wholistic wellness program (Wicklum et al., 2019), and improvements in $\dot{V}{O}_{2}$ max and self-reported moderate to vigorous PA (MVPA) (Lai et al., 2019). Only one program (Lavallée, 2007) used an Indigenous method to evaluate their intervention, a sharing circle methodology. Further, one author expressed explicit concern about the possible disconnect between study parameters and Indigenous culture (Daniel et al., 1999).

**Table 4:** Data extraction table

**Author (year)** **Location**	**Location**	**Title**	**Program/intervention goals**	**Program description**	**Evaluation summary**	**Barriers**	**Enablers (of success)**	**Limitations**	**Themes**
Brooks-Cleator and Giles (2016) Nova Scotia (rural)	Nova Scotia (rural)	Culturally Relevant Physical Activity through Elders in Motion: Physical Activity Programming for Older Aboriginal Adults in the Northwest Territories, Canada	1. To improve access to physical activity opportunities for Elders living in the NWT 2. To develop a training manual for program leaders and an accompanying video for Elder in Motion (EIM) 3. To deliver training of EIM to various health and recreational leaders, and students in the NWT	Program aimed at Indigenous Elders. Consists of 10 easy and effective exercises and a Balls and Balance activity. Group setting in recreation, community or health centres or can also be used in Elders’ homes. Flexible program, generally offered at same time each week. Article assessed cultural relevancy to communities involved	*n* = 7, program leaders only interviewed, no participants Semi-structured interviews and archival research. The themes demonstrated that some aspects of EIM were culturally relevant; however, some were not. Themes: (1) adaptations were made to EIM, but more can be done, and (2) more engagement with and focus on Elders are needed	Lack of support of program leaders and Elders overall (defunding of committees that establish Elder needs Lack of adequate prior engagement with local Elders Reinforcing colonial process by providing a standardized Western-based program	Cultural adaptation including language translation, representative pictures, videos, and music	Only able to comment on cultural relevancy of the program, no indicators of cultural safety as only program leaders were interviewed	Ensure cultural relevancy Ensure adequate resources Caution re: adapting a Western-medical approach
Ho et al. (2008) Ontario	Ontario	An Integrated Multi-Institutional Diabetes Prevention Program Improves Knowledge and Healthy Food Acquisition in Northwestern Ontario First Nations	1. To improve dietary choices and physical activity by increasing knowledge, self-efficacy, and attitudes about healthier behaviors 2. To increase opportunities to perform items above	Zhiiwapenewin Akino’maagewin: Teaching to Prevent Diabetes (ZATPD) program was a feasibility study of a 9-month integrated multi-institutional diabetes prevention program with school, store, and community components Intention to integrate program activities with existing activities	*n* = 113, feasibility study, quasi-experimental, pre/post evaluation ht, wt, PA (accelerometers), surveys, food frequency questionnaires No significant changes in PA or BMI Significant increase in knowledge and healthy food acquisition	Environmental barriers to outdoor PA; loose dogs, poor road and weather conditions, no exercise facilities Lack of personal time Intervention staff limited time and other staff inadequately resourced to support	Support for key stakeholder engagement at outset and responding and adapting to community needs Integration into the whole community Using children as change actors, store staff and community members to support integration Emphasis on cumulative daily, short bursts of activity	Short duration of intervention, low response rate of control community and non-random assignment of communities Lack of integration of components	Adequate duration of programming Cultural relevancy Outdoor environment not conducive to activity
Gray-Donald et al. (2000)	Quebec (on-reserve)	Intervening to reduce weight gain in pregnancy and gestational diabetes mellitus in Cree communities: An evaluation	1. To improve dietary intake during pregnancy 2. To optimize gestational weight gain, glycemic levels, and birth weight 3. To avoid unnecessary post-partum weight retention	1-year program utilizing Nutritionists and Cree health workers through strategies of modelling, skill training, contracting and self-monitoring with the activities of local radio broadcasts about healthy eating in pregnancy, pamphlets about nutritional choices and encouraging breast-feeding, supermarket tours and cooking demonstrations, exercise/walking groups, and individual counselling	*n* = 219, prospective, non-randomized intervention PA questionnaire, dietary questionnaires, maternal glucose, child-birth weight, and maternal weight No increases in physical activity noted	Desirability of being plump, and lack of desirability of PA in this particular community Walking outdoors can be very cold, and fitness classes were not seen as appropriate for pregnant women Intervention may not have been sufficiently intense	Nutritionists received training in cultural beliefs concerning diet, developed, and adapted local teaching aids and worked with a team of health care workers, including a community nutritionist working in the Cree villages. Cree health workers were hired in each of the 4 communities	Self-report measures	Cultural relevancy Physical environment conducive to being active Outdoor environment not conducive to activity
Dyck et al. (1998)	Saskatchewan (urban)	Preventing NIDDM among aboriginal people: is exercise the answer? Description of a pilot project using exercise to prevent gestational diabetes	1. To use exercise to prevent gestational diabetes 2. To complete a feasibility study	Eligible women are encouraged to attend a supervised exercise program three times per week until late pregnancy. Each session is 45 min long and consists of a 5-min warm-up, 30 min of aerobics, and a 10-min cool-down. The target heart rate is (220 − age × 0.7). Women instructed by Aboriginal assistant, a fitness instructor, and a registered nurse. High-impact sessions avoided	*n* = 5, Protocol description and broad comments about participation but challenges with contacting participants and collecting data results in lack of data to report Positive feeling about numbers of participants No follow-up/future studies could be found in the literature	View Aboriginal peoples are being over researched Inadequate referrals to program, challenges contacting and following up with participants	Partnership with the Aboriginal community, including the employment of an Aboriginal project workers and the performance of a needs assessment among pregnant Aboriginal women	Challenges with collection of data	Cultural relevancy
Higgins et al. (2004)	Vancouver Island	The Vancouver Island Race: a collaboration between the Saanich Peninsula Diabetes Prevention Project and Saanich Adult Education Centre [Steps Across Canada]	1. To encourage young adults to walk	4-week walking program with First Nations students, designed to promote a supportive, culturally sensitive environment for the uptake of physical activity Rewards for cumulative kms walked	News report type of article, no indication of ‘n’, no statistical analysis, walking distance measures and pre/post-questionnaire Walking helped to increase the positive aspects of their lives, as well as decrease the negative aspects by providing a break from routines, alleviating stress, and providing time to think and relax. Incredibly, the students not only achieved their goal of ‘walking’ the length of the Island, but they also actually surpassed it by ‘walking’ the length of the island two-and-half times	Participants found keeping logs to be ‘homework’, responded better to easier methods of reporting steps (not defined)	Teamwork and joy of walking	No analysis described	Context relevancy, capitalising on power of group work
Daniel et al. (1999)	Okanagan (rural), on-reserve	Effectiveness of community-directed diabetes prevention and control in a rural Aboriginal population in British Columbia, Canada	1. To reduce the prevalence of risk factors for NIDDM	24 months, participatory action research—activities included aerobics and gentle exercise classes, walking group, ‘100 Mile Club’, health events, cooking demonstrations, smoking cessation group, supermarket and restaurant tours, forums on diabetes, Diabetic Support Group and targeted education Media campaign Employment of a recreation coordinator by the Band Council to promote physical activity and create acceptable opportunities for exercise	*n* = 207 overall, but those who participated in all rounds of intervention are: Okanagan (pop-received intervention; *n* = 62), Spallumcheen (control; *n* = 27), Penticton (control; *n* = 16). Quasi-experimental, single evaluation Project yielded few changes in quantifiable outcomes. Activation of the intervention community was insufficient to enable individual and collective change 19 quantitative outcomes, significant changes in HbA1C (increased), mean systolic blood pressure decreased PA did not change Improvements occurred in the strength of peoples’ commitment to diabetes prevention. Evidence of these shifts includes support for hiring a Recreation Coordinator, greater participation on local sports teams and discussions of diabetes at gatherings (but should be interpreted with caution)	Program duration may have impacted results Project ending just as participants were starting to appreciate elements of it Lack of emphasis in interviews on elucidating indigenous ways of activating the community. It also suggests a failure to establish a community consensus on priorities, setting goals and outlining a division of responsibility	Community-based participatory action research	Nature of the interventions relate more to the health professional culture than to Aboriginal culture. Interventions did not unite theory or previous research with Aboriginal logic and concepts gleaned from qualitative analyses of diagnostic interviews	Duration Cultural relevancy and context
Anand et al. (2007)	Ontario (on-reserve)	A family-based intervention to promote healthy lifestyles in an aboriginal community in Canada	1. To support improved dietary and PA habits in families	Regular visit by health councillors to help families create dietary and PA goals Included nutrition education, recipes, grocery store tours, food prep classes	Intervention [29 families, 88 participants]; control [28 families, 86 participants] No change in PA (IPAQ scores) Sedentary behaviours reduced in the intervention (3.7 h to 3.1 h/day) vs. usual care (3.5 to 3.4 h/day) households—not statistically significant	Environmental/structural barriers to activity postulated; most of the roads on the Reserve are paved county roads designed for cars and trucks to travel at a speed of 80 km/h, no bicycle paths or sidewalks, safety concerns about walking at night and attacks from unleashed dogs, most adults must drive to work. While there are walking trails and running tracks at the schools on the Reserve, individuals have to drive to these locations to walk	Health councillors were Aboriginal Additional interventions added including filtered water, PA programming and educational events		Physical environment not conducive to activity
Sharma et al. (2010)	Nunavut (rural)	Addressing the public health burden caused by the nutrition transition through the Healthy Foods North (HFN) nutrition and lifestyle intervention programme	To reduce risk of chronic disease and improve dietary adequacy among Inuit/Inuvialuit in Nunavut and the Northwest Territories	12-month nutrition and PA intervention: pedometer challenge, coffee/tea/healthy breakfast; healthy snacks; healthy eating at home and traditional foods; healthier cooking and meal planning; getting enough vitamins and minerals	No clear sample size stated, for pedometer challenge enrolled 4–8 people per worksite, approx. 10–14 worksites. No PA results data presented. For non-PA results see Kolahdooz et al., 2014* below	Remoteness limits feasibility of interventions, availability and affordability of healthy food a barrier, appropriate environments for walking limited	Community-based participatory research, workshops supported community members to be involved in program development, execution and evaluation, fostered ownership and ensured relevancy to culture and context		
Kolahdooz et al. (2014)*	As above	As above	As above	As above	**Non-PA related** results which included: *n* = 332 (221 intervention, 111 control) Decrease in de-promoted foods, such as high-fat meats (− 27.9 g) and high-fat dairy products (− 19.8 g) among intervention communities (all *p* ≤ 0.05). Healthier preparation methods significantly increased (14.7%) in intervention communities	As above, and additionally educational material distributed via television and radio not accessible in remote areas	As above	Food frequency recall bias may impact results, response majority female participants (80–82%), lower response rate in certain communities	Nutrition education, HFN, can influence dietary patterns and food preparation methods
Foulds et al. (2011)	British Columbia, on-reserve	The effectiveness of community based physical activity interventions with Aboriginal peoples	1. To increase PA 2. To reduce cardiovascular disease risk	Hearts in Training community-based physical activity intervention for reducing CVD risk factors 13-week-long training programs, weekly group training sessions, with individual training 2 additional days per week Offered to over 242 Aboriginal groups around the province of British Columbia for individuals 12 years and older as training preparation for the Vancouver Sun Run 10 km event *Not clear evidence of cultural components but conducted and coordinated through local Aboriginal government therefore assume culturally relevant for communities choosing to do it	*n* = 273, M: 58, F: 215, 756 consented, self-report, pre/post PA scores improved, proportion of people meeting PA recommendations increased, walk/run, and run programs increase PA significantly	Not discussed in detail	Conducted and coordinated through local Aboriginal government and community representatives. Local Aboriginal health care professionals and volunteers assisted with testing, and participants were recruited through local First Nations band offices or Native Friendship Centres	Self-selected design and lack of a control group True attrition rate not determined Lack of objective measures of PA	
Darroch and Giles (2017)	Ontario, urban off-reserve	Conception of a resource: development of a physical activity and healthy living resource with and for pregnant urban First Nations and M√©tis women in Ottawa, Canada	1. To co-develop a resource (Celebrate Creation) helpful in promoting physical activity and reducing excessive weight gain during pregnancy Part of a larger research study aimed to understand and address micro and macro determinants of healthy pregnancies in First Nations and Metis women in Ottawa, Canada	Non-intervention—literature review and community engagement Interview of healthy service providers in Aboriginal communities Conduct focus groups with pregnant and post-partum First Nation and Metis women	*n* = 25 pregnant/post-partum FN + Metis women attended 3 focus groups (20) and semi-structured interviews (5) (8 attended follow-up focus group for resource development) *n* = 15 health/services providers attended interviews (7 attended follow-up focus group) Determined there was a need for tailored, culturally safe and positively framed First Nations and Metis resources, and online applications		Highlight of process of programmatic development embedded in community	Non-PA intervention, preparatory intervention only Did not discuss sharing circles or use of Indigenous methodology No assessment of the online application that was developed as a result of the study	Cultural and context relevancy Cultural safety
Wicklum (2019)	Alberta	Results of a Culturally Relevant, Physical Activity-Based Wellness Program for Urban Indigenous Women in Alberta, Canada	1. To increase PA 2. To increase nutrition education 3. To increase sense of community	8-week program with cultural (celebrations and sharing circles), PA and nutrition components Community-based research, local program facilitator hired and trained Supported by local Elder	*n* = 66, mixed methods, pre/post evaluation of 4 programs, no attrition rates indicated only research-defined attrition where analysis was only done on first timers and who attended a minimum of 3/8 weekly sessions (*n* = 49) Average weekly step count increased from week 1 to week 8 (*p* = 0.001) Improvements in confidence to participate in group exercise (*p* = 0.042) Greater than 50 achieved physical activity and nutrition goals No significant change in *n* = 21 semi-structured interviews—identify barriers and enablers Showed promise as a community-based model for supporting Indigenous women to improve their health Reasons for not attending included lack of childcare or transportation, health crises, including hospitalization and mental health issues, family crises and commitments, and other life stressors	Family networks non-existent or inadequate support, lack of childcare or transportation, limited social networks), financial and personal stress, including high cost of food, prohibitive costs of accessing facilities for PA and lack of familiarity or sense of security with PA infrastructure	Family networks (roles as mothers and grandmothers wanting to model for children and be well for family), socialization/sense of community among participants, shared accountability, multigenerational aspect, educational resources to bring to wider social network	Lack of control group Self-report of weekly step counts Short duration of intervention and study Challenges with survey completion	Cultural and context relevancy Cultural safety Holistic—all 4 components addressed
Lai et al. (2019)	British Columbia, rural, on-reserve	With every step, we grow stronger: The cardiometabolic benefits of an indigenous-led and community-based healthy lifestyle intervention	1. To improve health-related physical fitness within a rural and remote Indigenous community	13-week walking and healthy lifestyle counselling program, using tailored walking prescriptions and motivational interviewing principles when completing healthy lifestyle counselling	*n* = 15, pre/post program evaluation, Leisure Time Activity Questionnaire, accelerometer, 6-min walk test, grip strength, BIA, flexibility—sit-and-reach, WC measured Significant improvement in VO2 max, fitness group, individuals with the lowest cardiorespiratory fitness had the greatest improvements in VO2max, MVPA time spent in bouts of 30 min increased. Significant changes were also observed in self-reported MVPA, in which all fitness groups met international recommendations following the intervention. Discrepancies found between self-report and measured fitness	Finding community leaders who can participate Challenges with data collection, specifically wearing accelerometers	Culturally safe and Indigenous community engagement Community leaders participated in walking events Non-Indigenous professional who created PA prescriptions were trained in cultural respect and safety	Small sample size, lack of control group Discrepancies in reported versus measured fitness Cultural safety not evaluated	Cultural and context relevancy Cultural safety
Murdoch-Flowers et al. (2019)	Quebec, on-reserve	Understanding how Indigenous culturally-based interventions can improve participants’ health in Canada	1. To determine how the KSDPP interventions, as a suite of culturally based health promoting interventions, change how participants experience their life?	KS Diabetes Prevention Program—suite of interventions included (i) ‚ÄòHealthy Mind in a Healthy Body‚ Äô Workshops —included lectures on physical activity, (ii) Gentle and Standard Yoga classes, (iii) ‚ ÄòHaudenosaunee Foods Cooking‚ Äô Workshops, (iv) Fitness Program for Young Women/Mothers, and (v) Qigong classes	*n* = 18, 17 adult, female Kahnawake community members 1 Community Intervention Facilitator (CIF) designated as key informant Reported on ‘change processes’—learning physical activity, learning mind focusing and relaxation techniques	CIF leaving program	Culturally relevant Charismatic CIF with strong leadership qualities, able to communicate traditional Haudenosaunee values and knowledge	No objectives measures of physical health Small study size	Cultural and context relevancy Cultural safety Holistic—all 4 components addressed
Heffernan et al. (1999)	Haida Communities in Skidegate and Old Massett, British Columbia	The Haida Gwaii Diabetes Project: planned response activity outcomes	To reduce risk of diabetes	Haida Gwaii Diabetes Project, 3-phase project including chart review, focus groups, diabetes clinics, then traditional herbal medicine and traditional diet trial, and exercise classes 3/week for 15 months. Activities led by community members	*n* = 22, pre/post study. Significant decrease in weight, BMI, fasting blood glucose, total cholesterol and HbA1C, increased triglycerides and HDL *Getting Stronger* exercise program well attended. Exit group interviews reflected that the activities increased diabetes awareness and contributed to healthcare provider knowledge of community	Menu of activities was challenging to develop and execute for a small site. However, now the site is aware of which activities they may focus on in future	Operating principles of a project team adhered to well, was challenging but ensured appropriate representation of findings and built mutual respect and trust		
Klomp et al. (2003)	Saskatoon, Saskatchewan, urban	Description and evaluation of a prenatal exercise program for urban Aboriginal women	1. Promote regular physical activity in pregnant Indigenous women with previous GDM (gestational DM)	Free of charge, weekly 45-min prenatal fitness program with variety of exercises Led by certified exercise instructor, supported by Registered Nurse Coordinator, an Aboriginal Project Facilitator and Physiotherapist	*N* = 69 51% completed post-intervention program evaluation questionnaire for individuals who attended at least 4 sessions Researchers mention how women developed a ‘sense of solidarity and community among participants’	Sustainability—lack of local organizations’ interest in adoption of program	Incentives included designated social time and snacks Covered childcare, bus fare, and maternity bathing suits available Flexible drop-in time Ability to exercise at own comfort level Strong support from family members	Designed after program begun with limited evaluation purposes Attrition unreported	
Lavallée (2007)	Toronto, Ontario, urban	Physical activity and healing through the medicine wheel	Explore existing program’s impact using Indigenous research methods and framework	Martial arts classes at Indigenous Community Centre by Indigenous instructor Inclusion of traditional medicines, purification ceremonies, and gifts	Mixed methods *N* = 17 Evaluation using sharing circles 11/17 participated in symbol making Reported all four aspects of medicine wheel improved (emotional, mental, spiritual, physical)	None listed	Medicine Wheel as basis of health learning	No quantitative reporting of physical activity or weight-related outcomes Participants needed to have a baseline fitness Difficult to transfer directly to Indigenous communities outside urban context	

### Key Informant Interviews

Fifteen key informant interviews were completed of individuals representing different types of organizations.

There were six PA-based programs for Indigenous women identified nationally—the focus of this study. Five key informants interviewed from these programs were completed, excluding a Makoyoh’sokoi informant to avoid bias. Two of the programs started out as private, for-profit ventures but later were forced to pursue government relationships; all others were not for-profit, and government or grant funded. The programs were/are one each in British Columbia (short-term walking programs with separate cultural events), Alberta (comprehensive 15-week wellness program—Makoyoh’sokoi), Saskatchewan (drop-in wellness, and training for fitness instructors), Manitoba (mainly short-term walking programs, challenges with separate cultural elements), Ontario (fitness classes and separate groups incorporating cultural elements), and Quebec (on-the-land activities, family focused and comprehensive). ‘Comprehensive’ implies the inclusion of nutrition, cultural, or spiritual elements. Key informants from PA-based programs for Indigenous women identified the importance of consistent programming, identifying champions from community to be involved in programming and issues with childcare provision, adequate funding, and inadequate participant and organization resources for elements such as healthy foods, equipment, and infrastructure.

Three informants represented organizations with programming that was not PA-based but was specific to the health of Indigenous women, and seven represented organizations with general expertise in PA and Indigenous populations.

All the programs were government or agency funded; two had a combination of paid and community-sponsored programming elements. For all programs, financial constraint of individuals was identified as a major challenge. Interview results are tabulated in Supplementary Table B.

### Collation of scoping review and key informant interviews

Table 5 outlines the themes for both promising practices, and intervention or organizational deficits/barriers, as identified from the scoping review (17 articles, 16 interventions) and key informant interviews (*n* = 15). ‘Additional themes’ beyond these, but that provide valuable insight, were also included.

**Table 5:** Promising practices and challenges of programming identified in scoping review and through key informant interviews

**Promising practices**	
Involve community in design and operations. Identify community champions or role models to support the project	“*People should be paid for facilitating and board work; peer leaders get honoraria for time, transportation and childcare; advisory board positions seen as a job*” “*Marketing is a process; you must be willing to go in to the community and introduce yourself and build relationships*”
Ensure cultural relevancy and safety. Acknowledge presence of many different Indigenous cultures and traditions in any one area, avoid pan-Indigenisation. Use Indigenous languages	
Value Indigenous ways of knowing and teachings	“*The Elders always ground us by stating ‘every meeting today will affect 7 generations*’”
Position health positively, avoid deficit modelling	
Include elements of health beyond PA, these elements are valued by participants, e.g., traditional ceremony and foods, sharing circle opportunities	“*I try to get people in tune to ‘self’*” “*Having music is key to the program’s success, the wholistic nature is well received*” “*Dance and Drum teaches respect and* [the] *7 sacred teachings, how to find that balance within that medicine, and to make that structure for themselves—how to realize that they are worthy*”
Be cautious when using or adopting a Western medical model	
Group intervention model is important	“*Participants want to be part of something*” “*Making and having a connection with people is the incentive for them to want to keep coming back*”
Facebook and word of mouth useful, and most common, for marketing	
Offer prizes to encourage participation	
Walk or walk/run programs are common and can increase PA significantly	
Program consistency week over week and year over year is important	“*Consistency of the programming is important for attendance, the program being reliable is meaningful* [to them]”
**Challenges—organizational**	
Lack of programming for adult women Inadequate resources, personnel and financial	
Inadequate resources to evaluate programming. Evaluations frequently limited to basic demographics, attendance, and satisfaction commentary only	“*Some people view recreation sector as a summer job or part-time, misconception; but it is a career//lots of departures in rec sector: recreation personnel in communities take on broad job descriptions – low support but high demands; creates a lot of people thinking it’s not worth it*”
Short duration of programs—longer durations are needed to achieve impact (notably, one of the longer programs did not show improved outcomes)	
Disconnect between goals of funders and community	“[Important for partners] *to truly have the interests of the program in mind*” “[Our location] *is a very special place, we are very fortunate to have the resources that we have and the government is structured differently from others jurisdictions… functions more as coalition and partnership with Indigenous governments… hopeful… resurgence of re-investment into cultural programming and mental health programming, like supporting wellbeing … you can promote PA until blue in the face but if it clashes with local culture… people know what is healthy but opportunities must align with cultures that are healthy and that are important for them*”
**Challenges—participant specific**	
Financial constraints of target participants	“*Participants and community in general* [are challenged] *to meet threshold of Canadian food guide*”
Positioning health status from a negative viewpoint or deficit was viewed negatively – preference for positivist/strength-based view	
Outdoor environment perceived as unsafe and negatively impacting walkability, and therefore a barrier to PA	
Vulnerability of participants needs to be deeply understood	“*Vulnerable population already moves a lot out of necessity, fitness can be triggering, especially fitness classes*”
**Additional themes**	
On-the-land activities are important	
Sports programming for children, youth, and adults is more prevalent than non-sport physical activities Mothers and fathers (women and men) have different programming needs	“*Moms and dads group separately. Moms wanted to be alone, and dads wanted things to do with the kids*”
Programming that begins with youth in community, can lead to the recognition of the need for adult programming	
In relation to the impact of the COVID-19 pandemic—virtual adaptation holds potential to reach more individuals	
Photovoice and interviews well received evaluative elements	“*Semi-structured interviews and photovoice, well received, healing is not linear*”

## Discussion

This environmental scan builds upon our earlier work on PA-based wellness programs for Indigenous women (Wicklum et al., 2021). This work highlights the dearth of wholistic interventions addressing Indigenous women’s health in Canada, from both published and unpublished literature, and through key informants. Key informant interviews confirmed that PA-based programming for Indigenous children and access to organized sports is more prevalent than PA programming for women. There is a clear gap in programming focussed on non-sport PA for Indigenous women in Canada.

Several elements for consideration when creating wellness (illness prevention) interventions for Indigenous women were identified: the value of incorporating Indigenous ways of knowing; the need to use a community-based approach; avoiding pan-Indigeneity, respecting each Nation or communities’ uniqueness and differences; using a positivist lens versus deficit lens; and the need for adequate financial, and other, resources and support for programming.

Embedding elements of culture in programming is well received, from an educational standpoint, simply gaining an understanding of what culture means and specific practices can be important to individuals, and from a spiritual standpoint it provides grounding for individuals wanting to reconnect or affirm their connection to culture. “*The Elders always ground us by stating every meeting today will affect 7 generations*” (key informant). The wholistic view of health, that is traditional of many Indigenous cultures, was recently reviewed by Miles et al. (Miles et al., 2023). Elder Pablo Russell, when teaching ‘The Buffalo Way’ describes how the ‘mental’ (the mind, making decisions, reasoning) and ‘physical’ aspect of health dominate for most people in the present day, and to achieve balance, ‘spiritual’ and ‘emotional’ aspects, must also be addressed (Russell, 2016). This study reaffirmed Elder Russell’s point that outcomes beyond ‘physical’ (anthropometric or fitness) changes are very important. Changes in spiritual and emotional aspects of health are challenging to measure and may be better suited to qualitative, and specifically, Indigenous methodologies. One key informant stated that “*semi-structured interviews and photovoice*, [were] *well received, healing is not linear*”, and these methods may reflect non-linear changes better. Further, other researchers have described the many benefits of Indigenous methodologies, noting these are still emerging and rigor and consistency in methods needs to be applied (Kennedy et al., 2022).

It is well understood that a community-based approach to developing programs is essential and all but one intervention in this study was community-based. Creating interventions with community member involvement and control helps ensure they are well adapted to the unique needs of individuals and their culture and traditions within the community context. This study suggested that there should be caution when applying or adapting a Western medical model for an Indigenous intervention; it was thought to adversely affect programming for one intervention (Brooks-Cleator & Giles, 2016). Indigenous culture is typically one where relations are of high importance. This study helps to reinforce this notion, as the group (developing a sense of community support) exercise programs led to health improvements more than the individual exercise programs. Social cognitive theory, that describes relationships among social and psychological factors determine a health behaviour, supports this notion. The realist analysis of Makoyoh’sokoi described two of three positive outcomes of their program as related to ‘group’ or ‘community’ (Wicklum et al., 2023). The program was found to work by ‘support[ing] self-actualization through collective effort’ and ‘provid[ing] social and cultural supports’. Moreover, Richmond also concluded social support as a ‘strong determinant of thriving health, particularly among [Indigenous] women’ (Richmond 2007).

Pan-Indigeneity arose as a potential concern during program design. In many locations, there can be multiple different Indigenous cultures with different traditions and programming should acknowledge and respect this, working to ensure program contents reflect the uniqueness versus drive programming towards a homogenized Indigenous approach.

Another important program design element identified by this scan included positioning Indigenous women as resilient, using a positivist lens reinforces individual value and decreases emphasis on weaknesses. It acknowledges the Indigenous perspective of wholistic health and the understanding of the severely negative impact of colonization on health but does not assume weakness and negativity but rather resiliency and strength. McGuire-Adams takes this further in Indigenous Feminist Gikendaasowin (Knowledge) – Decolonization through PA (McGuire-Adams, 2020). She writes about the importance of ‘Indigenous peoples supporting each other to overcome the negative ramifications of colonialism on their bodies, specifically regarding ill health’. She writes how high levels of PA were traditionally commonplace, and represented power and resiliency, and how in present day women can return to intense PA as a means of decolonizing.

Inadequate financial resources, of community organizations running programs and participants themselves, were identified as a significant barrier to programming. Funding for programming was cited as a challenge by many key informants. Two of our key informants who began with the typical for-profit, client-based programming had to convert to be funded by larger organizations who obtained government funding in order to remain viable. All other programs were government funded, either directly or indirectly. Limited budgets meant inadequate staff and funds to complete robust evaluations as funding prioritized the actual programming elements. In 2022, Indigenous women earned 8% less than Indigenous men, and both Indigenous women and men earn 20.1% and 12.3% less than Canadian-born men (Drolet & Mardare Amini, 2023). There has been some recent improvement in finances for Indigenous peoples with fewer Indigenous peoples living in a lower income situation (Government of Canada, 2023). Moreover, budget investment in Indigenous sport leadership and culturally relevant sport programming since 2017 and in March 2024 and a new funding stream was launched by the federal government to ‘support the improved mental and physical health of Indigenous women and girls and 2SLGBTQI + people’. For Indigenous populations, health prevention programming remains as a priority of both Indigenous and non-Indigenous governments, and investments need to continue to be made.

The limitation of this environmental scan includes the fact that wellness interventions may have existed pre-COVID-19 and ceased to exist during the pandemic and therefore could not be found. Other programs may exist that we were unaware of or were not searchable in published or grey literature, or through the key informant interviews. Key informants were asked to respond to questions based on experience versus opinion where appropriate, and due to the lack of programming specific to Indigenous women, only five informants had hands-on experience. Furthermore, we did not interview or survey the actual participants of the various programs and, therefore, do not have the participant lens represented except as it was through the published articles.

Our methods revealed the importance of applying a comprehensive search strategy when completing reviews that relate to Indigenous populations. This scoping review was the first to utilize new and more inclusive (e.g., on-the-land activities) search terms for PA and more extensive search terms for ‘Indigeneity’. During the time that this study was completed, the University of Alberta established an Indigenous search term strategy which is comprehensive and mimics our final search (Campbell et al., 2013). Overall, the methodology, specifically doing key informant interviews, was effective in identifying important themes which would not have happened using published literature only. Our findings align with the research priority of ‘identifying characteristics of effective sport and PA interventions’ as defined by Bélanger et al. (2022).

## Conclusion

The findings of this scan underscore a notable gap in PA-based programming tailored for adult Indigenous women throughout Canada. The voices of the key informants were united and resolute in their desire to establish such initiatives. This acknowledgment and work create a pathway forward, highlighting the imperative need for concerted efforts to develop and implement wholistic programs that prioritize the physical, mental, emotional, and spiritual well-being of Indigenous women. Recent funding announced by the federal government, Sport for Social Development in Indigenous Communities (SSDIC) Stream 3, will support this work, but other levels of government and organizations will also need to prioritize this work to ensure Indigenous women a healthy future.

## Contributions to knowledge

What does this study add to existing knowledge?

- This study highlights the lack of physical activity (PA)–based programming for Indigenous women. Further, that programming to date, and organizational focus, generally addresses youth PA and adult sports that are more tailored to men.
- Existing knowledge emphasizes the importance of strong community engagement and cultural safety when developing programming. This study reinforces those principles and adds the value of walk and walk/run programming for this population, and the importance of a wholistic approach to health (versus solely addressing PA), consistency and reliability of programming week to week and year over year, and stable resourcing (financial and personnel).

What are the key implications for public health interventions, practice, or policy?

- PA interventions to improve the health of Indigenous women should be prioritized to address high rates of disease, aligning with the TRC Calls to Action # 87–91. These interventions should be wholistic in design (include elements of mental, spiritual, and emotional well-being), grounded in regional culture, and well-funded to support consistency and reliability to provide a backbone of stability that will enhance gradual uptake by Indigenous women as trust is built with the programmer.
- Indigenous methods were rarely used to evaluate programming, and efforts should be made to change this.

## Additional questions

- 1.Are First Nations, Inuit, Métis, and/or Indigenous Peoples or populations a focus of this submission? Yes
- 2.If yes, were the relevant Indigenous Peoples or populations engaged in the study and/or preparation of this submission?Yes
- 3.If yes, please summarize how the relevant Indigenous Peoples or populations have been engaged as individuals and collectively in the study and/or preparation of this submission (2000-character limit).

There are Indigenous Peoples involved throughout this study; as indicated in the paper, an Advisory Group (AG) with an Elder, a past Facilitator, and a community member who are all Indigenous, and a group of that are called ‘Community Leads’ for our Makoyoh’sokoi Project are all Indigenous and represent their respective communities. Lindsay Crowshoe, Loretta Tuttauk, and Tia Black are members of our research team and are also Indigenous. The AG and CL were involved in decision-making about the study design and the thematic analysis of both the scoping review and the key informant interview components of the study.

## Supplementary Information

Below is the link to the electronic supplementary material.Supplementary Material 1 (DOCX 24.6 KB)

Below is the link to the electronic supplementary material.Supplementary Material 2 (DOCX 37.6 KB)

## Data Availability

All data from search and informant interviews is available in supplementary tables.
