# Supplementary material for: Physical activity–based, wholistic, wellness interventions for Indigenous women in Canada: An environmental scan to identify programs and promising practices
Source: Can J Public Health. 2025 Nov 6;117(4):610–33. doi: 10.17269/s41997-025-01091-9 (PMC13534398; doi:10.17269/s41997-025-01091-9)
Supplement: Supplementary file 1 [file 41997_2025_1091_MOESM1_ESM.docx]

**Supplementary Table A: Search Strategies Used**

**Table A1: Indigenous search filters used from University of Alberta library. ***

*Filters adapted for other databases used, version used is October 2021.

| **Database** | **Search Terms** |
| --- | --- |
| **OVID MEDLINE** | (exp Indians, North American/ or exp Inuits/ or exp Health Services, Indigenous/ or exp Ethnopharmacology/ or Athapaskan.mp. or Saulteaux.mp. or Wakashan.mp. or Cree.mp. or Dene.mp. or Inuit.mp. or Inuk.mp. or Inuvialuit*.mp. or Haida.mp. or Ktunaxa.mp. or Tsimshian.mp. or Gitsxan.mp. or Nisga'a.mp. or Haisla.mp. or Heiltsuk.mp. or Oweenkeno.mp. or Kwakwaka'wakw.mp. or Nuu chah nulth.mp. or Tsilhqot'in.mp. or Dakelh.mp. or Wet'suwet'en.mp. or Sekani.mp. or Dunne-za.mp. or Dene.mp. or Tahltan.mp. or Kaska.mp. or Tagish.mp. or Tutchone.mp. or Nuxalk.mp. or Salish.mp. or Stl'atlimc.mp. or Nlaka'pamux.mp. or Okanagan.mp. or Sec wepmc.mp. or Tlingit.mp. or Anishinaabe.mp. or Blackfoot.mp. or Nakoda.mp. or Tasttine.mp. or Tsuu T'inia.mp. or Gwich'in.mp. or Han.mp. or Tagish.mp. or Tutchone.mp. or Algonquin.mp. or Nipissing.mp. or Ojibwa.mp. or Potawatomi.mp. or Innu.mp. or Maliseet.mp. or Mi'kmaq.mp. or Micmac.mp. or Passamaquoddy.mp. or Haudenosaunee.mp. or Cayuga.mp. or Mohawk.mp. or Oneida.mp. or Onodaga.mp. or Seneca.mp. or Tuscarora.mp. or Wyandot.mp. or Aboriginal*.mp. or Indigenous*.mp. or Metis.mp. or red road.mp. or "on reserve".mp. or off-reserve.mp. or First Nation.mp. or First Nations.mp. or Amerindian.mp. or (urban adj3 (Indian* or Native* or Aboriginal*)).mp. or ethnomedicine.mp. or country food*.mp. or residential school*.mp. or ((exp Medicine, Traditional/ or traditional medicine*.mp.) not Chinese.mp.) or exp Shamanism/ or shaman*.mp. or traditional heal*.mp. or traditional food*.mp. or medicine man.mp. or medicine woman.mp. or autochtone*.mp. or (Native* adj1 (man or men or women or woman or boy* or girl* or adolescent* or youth or youths or person* or adult or people* or Indian* or Nation or tribe* or tribal or band or bands)).mp.) and (exp Canada/ or (Canad* or British Columbia or Colombie Britannique or Alberta or Saskatchewan or Manitoba or Ontario or Quebec or Nova Scotia or New Brunswick or Newfoundland or Labrador or Prince Edward Island or Yukon Territory or NWT or Northwest Territories or Nunavut or Nunavik or Nunatsiavut or NunatuKavut).mp.) |
| **OVID EMBASE** | (Indigenous People/ or American Indian/ or First Nation/ or Metis/ or Eskimo/ or Inuit/ or Indigenous Health Services/ or exp Ethnopharmacology/ or (Algonquin or Anishinaabe or Assiniboine or Athapaskan or Blackfoot or Cayuga or Chippewa or Cree or Dakelh or Dene or Dunne-za or Gitsxan or Gwich'in or Haida or (Han not (Chinese or China or Tibet*)) or Haisla or Heiltsuk or Haudenosaunee or Kwakwaka'wakw or Inuit or Inuk or ((Iroquios or Iroquoian) not (homeobox or corn)) or Kaska or Ktunaxa or Nlaka'pamux or Nakoda or Nakota or Nuxalk or Maliseet or Meits or Mi'kmaq or Micmac or Mohawk or Nipissing or Nisga'a or Nuu chah nulth or jibwa or Ojibwe or Ojibway or Oji-Cree or Okanagan or Oneida or Onodaga or Oweenkeno or Passamaquoddy or Potawatomi or Salish or Saulteaux or Sec wepmc or Sekani or Seneca or Stl'atlimc or Tagish or Tasttine or Tahltan or Tlingit or Tsimshian or Tsilhqot'in or Tsuu T'inia or Tuscarora or Tutchone or Wakashan or Wyandot).mp. or Aboriginal* or Indigenous* or red road or "on reserve" or off-reserve or First Nation or First Nations or Amerindian or (urban adj3 (Indian* or Native* or Aboriginal*)) or ethnomedicine or country food* or residential school* or ((exp Medicine, Traditional/ or traditional medicine* ) not Chinese ) or exp Shamanism/ or shaman* or traditional heal* or traditional food* or medicine man or medicine woman or autochtone* or (Native* adj1 (american or man or men or women or woman or boy* or girl* or adolescent* or youth or youths or person* or adult or people* or Indian* or Nation or tribe* or tribal or band or bands)) ) and (exp Canada/ or (Canad* or British Columbia or Colombie Britannique or Alberta or Saskatchewan or Manitoba or Ontario or Quebec or Nova Scotia or New Brunswick or Newfoundland or Labrador or Prince Edward Island or Yukon Territory or NWT or Northwest Territories or Nunavut ) ) or (Canadian Aboriginal/ NOT oriental medicine/) or (Innu or Inuvialuit* or Nunavik or Nunatsiavut or NunatKavut or Wet'suwet'en ) |
| **EBSCO CINAHL** | ( ( (MH "Eskimos") OR (MH "Native Americans") OR (MH "Indigenous Peoples+") or (MH "Health Services, Indigenous") or (MH "Indigenous Health") or (MH “Ethnopharmacology”) or Athapaskan or Saulteaux or Wakashan or Cree or Dene or Inuit or Inuk or Inuvialuit* or Haida or Ktunaxa or Tsimshian or Gitsxan or “Nisga'a” or Haisla or Heiltsuk or Oweenkeno or “Kwakwaka'wakw” or “Nuu chah nulth” or “Tsilhqot'in” or Dakelh or “Wet'suwet'en” or Sekani or “Dunne-za” or Dene or Tahltan or Kaska or Tagish or Tutchone or Nuxalk or Salish or “Stl'atlimc” or “Nlaka'pamux” or Okanagan or “Sec wepmc” or Tlingit or Anishinaabe or Blackfoot or Nakoda or Tasttine or “Tsuu T'inia” or “Gwich'in” or Han or Tagish or Tutchone or Algonquin or Nipissing or Ojibwa or Potawatomi or Innu or Maliseet or “Mi'kmaq” or Micmac or “Mic mac” or Passamaquoddy or Haudenosaunee or Cayuga or Mohawk or Oneida or Onodaga or Seneca or Tuscarora or Wyandot or Aboriginal* or Indigenous* or Metis or “red road” or "on reserve" or “off reserve” or “First Nation” or “First Nations” or Amerindian or (urban N3 (Indian* or Native* or Aboriginal*)) or ethnomedicine or “country food*” or “residential school*” or ((MH “Medicine, Traditional”) or "traditional medicine*" ) not Chinese ) or ( MH“Shamanism”) or shaman* or “traditional heal*” or “traditional food*” or “medicine man” or “medicine woman” or autochtone* or (Native* N1 (American* or man or men or women or woman or boy* or girl* or adolescent* or youth or youths or person* or adult or people* or Indian* or Nation or tribe* or tribal or band or bands)) ) ) AND ( ( (MH “Canada+”) or (Canad* or “British Columbia” or “Colombie Britannique” or Alberta or Saskatchewan or Manitoba or Ontario or Quebec or “Nova Scotia” or “New Brunswick” or Newfoundland or Labrador or “Prince Edward Island” or “Yukon Territory” or NWT or “Northwest Territories” or Nunavut or Nunavik or Nunatsiavut or NunatuKavut)) ) |

**Table 1B: Overview of population and physical activity key terms***

*Adapted key terms to use appropriate “AND Canad*” as only location-related search term.

| Current search population key terms (excluding overlap) | Pelletier population key terms | Current search physical activity key terms (excluding overlapping terms) | Pelletier physical activity key terms |
| --- | --- | --- | --- |
| native people*  on-reserve  off-reserve  North American  Arctic Region*  AmericanNative*  Algonquin  aleut*  anishinabek  Anishnabek  Chipewyan  cree  Dene  eskimo*  gitksan  huron  Innu  inuktitut  inuk  inupiat*  iqaluit  iroquois  kalaallit*  Kawawachikamach  "Kawawachikamach Québec"  kahnawa:ke  kitikmeot  kitimat  kivalliq  Kwakiutl  manitoulin  metis  miawpukek  micmac  mi'kmaq  mohawk  mushkegowuk  naskapi  nisga'a  nakoda  nakota  oji-cree  ojibway  oki  opaskwayak  pauktuutit  Qikiqtani  qayuqtuvik  "Rankin Inlet"  sekon  sioux  tungasugit  tuttarvingat  "Vuntut Gwitchin"  akwesasne  athabasca  "Canadian Arctic"  "Chesterfield Inlet"  Deline, Northwest Territories  Eeyou Istchee  inukjuak  igluligaarjuk  inuvialuit  ivujivik  "James Bay"  kuujjuaq  "Mackenzie River Basin"  mistissini  nain  nemaska  nunatsiavut  nunavut  nunavik  nutaqqavut  Oujé-Bougoumou Ouje-Bougoumou  shubenacadie  "Slave Lake"  subarctic  wikwemikong  British Columbia  Columbie Britannique  Alberta  Saskatchewan  Manitoba  Ontario  Quebec  Nova Scotia  New Brunswick  Newfoundland  Labrador  Prince Edward Island  Yukon Territory  NWT  Northwest Territories  AND Canad* | (first nations or indigenous or aboriginal or native peoples or indian or eskimo or inuit or metis) AND canada (changes for each database) AND adult | exercise*  sport*  walk*  Obesity  "obesity prevention"  "fitness"  "physical activity intervention"  "active living"  "physical literacy"  "community engagement"  "community participation"  lacrosse  wrestling  fishing  Canoeing  archery  dance*  traditional games  hunt*  round dance  run*  jog*  Leisure  recreation  food gathering  Physical activit*  Hoop Dancing | activit*  aerobic exercise  anaerobic exercise  aquatic exercise  early Intervention  exercise  exercise test  exercise tolerance  physical activity  fitness  intervention study  strength training  cardiovascular training  sports  physical  stretching exercise  team sport  treadmill exercise |

**February 2022 note: for inclusivity, decided to remove (adult* OR women) search parameter and only keep PA, Indigenous and Canada terms (some identified papers did not explicitly state adult* or women in title/Abs but were highly relevant to our search, and thus were not being picked up when adult* or women was included in search parameters); adult was ORIGINALLY included, women was added Jan 2022 but both taken off in February.

**Table 1C: Example of stepwise search strategy for Web of Science***

*Adapted for other databases used.

| Pelletier original search (1990-2017) | TS=(("Activit*" OR "Aerobic exercise" OR "Anaerobic exercise" OR "Aquatic exercise" OR "Early Intervention" OR "exercise" OR "Exercise test" OR "Exercise tolerance" OR "Intervention study" OR "Strength training" OR "Cardiovascular training" OR "Sports " OR "Physical" OR "Stretching exercise" OR "Team sport" OR "Treadmill exercise" OR "fitness" OR "strength training" OR "cardiovascular training") AND ("first nations" OR "indigenous" OR "aboriginal" OR "native peoples" OR "indian" OR "eskimo" OR "inuit" OR "metis") AND (“canada" OR "canadian" OR "canadians") |
| --- | --- |
| Additional physical activity terms included | TS=("Activit*" OR " Aerobic exercise" OR "Anaerobic exercise" OR "Aquatic exercise" OR "Early Intervention" OR "exercise" OR Exercise test" OR "Exercise tolerance" OR "Intervention study" OR "Strength training" OR "Cardiovascular training" OR "Sports " OR "Physical" OR "Stretching exercise" OR "Team sport" OR "Treadmill exercise" OR "fitness" OR "strength training" OR "cardiovascular training" OR "lacrosse" OR "wrestling" OR "fishing" OR "canoeing" OR "archery" OR "dance*" OR "traditional games" OR "hunt*" OR "round dance" OR “run*” OR “jog*” OR “leisure” OR “recreation” OR "food gathering" OR "physical activit*" OR "hoop dancing" ) AND ("first nations" OR "indigenous" OR "aboriginal" OR "native peoples" OR "indian" OR "eskimo" OR "inuit" OR "metis") AND (“canada" OR "canadian" OR "canadians") |
| Both physical activity and Indigenous terms supplemented | TS=(("Activit*" OR "Aerobic exercise" OR "Anaerobic exercise" OR "Aquatic exercise" OR "Early Intervention" OR "exercise" OR “Exercise test" OR "Exercise tolerance" OR "Intervention study" OR "Strength training" OR "Cardiovascular training" OR "Sports " OR "Physical" OR "Stretching exercise" OR "Team sport" OR "Treadmill exercise" OR "fitness" OR "strength training" OR "cardiovascular training" OR "lacrosse" OR "wrestling" OR "fishing" OR "canoeing" OR "archery" OR "dance*" OR "traditional games" OR "hunt*" OR "round dance" OR “run*” OR “jog*” OR “leisure” OR “recreation” OR "food gathering" OR "physical activit*" OR "hoop dancing") AND ("first nations" OR "indigenous" OR "aboriginal" OR "native peoples" OR "indian" OR "eskimo" OR "inuit" OR "metis" OR "on-reserve" OR "off-reserve" OR "North American" OR "Arctic Region*" OR "American Native*" OR "Algonquin" OR "aleut*" OR "Chipewyan" OR "cree" OR "Dene" OR "eskimo*" OR "gitksan" OR "huron" OR "Innu" OR "inuktitut" OR "inuk" OR "inupiat*" OR "iqaluit" OR "iroquois" OR "kalaallit*" OR "Kawawachikamach" OR "kahnawa:ke" OR "kitikmeot" OR "kitimat" OR "kivalliq" OR "Kwakiutl" OR "manitoulin" OR "miawpukek" OR "micmac" OR "mi'kmaq" OR "mohawk" OR "mushkegowuk" OR "naskapi" OR "nisga'a" OR "nakoda" OR "nakota" OR "oji-cree" OR "ojibway" OR "oki" OR "opaskwayak" OR "Qikiqtani" OR "Rankin Inlet" OR "sioux" OR "Vuntut Gwitchin" OR "akwesasne" OR "athabasca" OR "Canadian Arctic" OR "Chesterfield Inlet" OR "Deline" OR "Northwest Territories" OR "Eeyou Istchee" OR "inukjuak" OR "inuvialuit" OR "ivujivik" OR "James Bay" OR "kuujjuaq" OR "Mackenzie River Basin" OR "mistissini" OR "nain" OR "nemaska" OR "nunatsiavut" OR "nunavut" OR "nunavik" OR "nutaqqavut" OR “Ouje-Bougoumou” OR "shubenacadie" OR "Slave Lake" OR "subarctic" OR "wikwemikong") AND (“canada" OR "canadian" OR "canadians") |

**Table 1D**: **Full example of Scopus search strategy***

*Adapted for remaining databases searched but not included in this table. Overall strategy was to combine (1) population and (2) physical activity search terms with (4) appropriate “AND Canad*” location key word(s), then (5) add time constraints. There were no language limitations.

| **Scopus** | title-abs-key(("first nations" OR indigenous OR aboriginal OR "native peoples" OR indian OR eskimo OR inuit OR metis OR on-reserve OR off-reserve OR "North American" OR "Arctic Region*" OR "American Native*" OR Algonquin OR aleut* OR Chipewyan OR cree OR Dene OR eskimo* OR gitksan OR huron OR Innu OR inuktitut OR inuk OR inupiat* OR iqaluit OR iroquois OR kalaallit* OR Kawawachikamach OR kahnawa:ke OR kitikmeot OR kitimat OR kivalliq OR Kwakiutl OR manitoulin OR miawpukek OR micmac OR mi'kmaq OR mohawk OR mushkegowuk OR naskapi OR nisga'a OR nakoda OR nakota OR oji-cree OR ojibway OR oki OR opaskwayak OR Qikiqtani OR "Rankin Inlet" OR sioux OR "Vuntut Gwitchin" OR akwesasne OR athabasca OR "Canadian Arctic" OR "Chesterfield Inlet" OR Deline OR "Northwest Territories" OR "Eeyou Istchee" OR inukjuak OR inuvialuit OR ivujivik OR "James Bay" OR kuujjuaq OR "Mackenzie River Basin" OR mistissini OR nain OR nemaska OR nunatsiavut OR nunavut OR nunavik OR nutaqqavut OR “Ouje-Bougoumou” OR shubenacadie OR "Slave Lake" OR subarctic OR wikwemikong) **AND** (exercise or "physical activity" or fitness or "aerobic training" or "strength training" or "cardiovascular training" or workout or active or movement or lacrosse or wrestling or fishing or canoeing or archery or dance* or "traditional games" or hunt* or "round dance" or run* or jog* or leisure or recreation or "food gathering" or "physical activit*" or "hoop dancing") **AND** (canada or canadian or canadians) **AND** (adult) **AND** ( LIMIT-TO ( PUBYEAR,2021) OR LIMIT-TO ( PUBYEAR,2020) OR LIMIT-TO ( PUBYEAR,2019) OR LIMIT-TO ( PUBYEAR,2018) OR LIMIT-TO ( PUBYEAR,2017) )) |
| --- | --- |
| **OVID Medline** | (Activit* or Aerobic exercise or Anaerobic exercise or Aquatic exercise or Early Intervention or exercise or Exercise test or Exercise tolerance or Intervention study or  Strength training or Cardiovascular training or Sports or Physical or Stretching exercise or Team sport or Treadmill exercise or fitness or strength training or  cardiovascular training or lacrosse or wrestling or fishing or canoeing or archery or dance* or traditional games or hunt* or round dance or run* or jog* or leisure or  recreation or "food gathering" or "physical activit*" or "hoop dancing").mp. **AND**  ("First nations" or indigenous or aboriginal or "native peoples" or indian or eskimo or inuit or metis).mp. or exp Indians, North American/ or exp Inuits/ or exp Health  Services, Indigenous/ or exp Ethnopharmacology/ or Athapaskan.mp. or Saulteaux.mp. or Wakashan.mp. or Cree.mp. or Dene.mp. or Inuit.mp. or Inuk.mp. or Inuvialuit*.mp. or Haida.mp. or Ktunaxa.mp. or Timshian.mp. or Gitsxan.mp. or Nisga'a.mp. or Haisla.mp. or Heiltsuk.mp. or Oweenkeno.mp. or Kwakwaka'wakw.mp. or Nuu chah nulth.mp. or Tsilhqot'in.mp. or Dakelh.mp. or Wet'suwet'en.mp. or Sekani.mp. or Dunne-za.mp. or Dene.mp. or Tahltan.mp. or Kaska.mp. or T  agish.mp. or Tutchone.mp. or Nuxalk.mp. or Salish.mp. or Stl'atlimc.mp. or Nlaka'pamux.mp. or Okanagan.mp. or Sec wepmc.mp. or Tlingit.mp. or Anishinaabe.mp. or Blackfoot.mp. or Nakoda.mp. or Tasttine.mp. or Tsuu T'inia.mp. or Gwich'in.mp. or Han.mp. or Tagish.mp. or Tutchone.mp. or Algonquin.mp. or Nipissing.mp. or Ojibwa.mp. or Potawatomi.mp. or Innu.mp. or Maliseet.mp. or Mi'kmaq.mp. or Micmac.mp. or Passamaquoddy.mp. or Haudenosaunee.mp. or Cayuga.mp. or Mohawk.mp. or Oneida.mp. or Onodaga.mp. or Seneca.mp. or T  uscarora.mp. or Wyandot.mp. or Aboriginal*.mp. or Indigenous*.mp. or Metis.mp. or red road.mp. or "on reserve".mp. or off-reserve.mp. or First Nation.mp. or First Nations.mp. or Amerindian.mp. or (urban adj3 (Indian* or Native* or Aboriginal*)).mp. or ethnomedicine.mp. or country food*.mp. or residential school*.mp. or ((exp Medicine, Traditional/ or traditional medicine*.mp.) not Chinese.mp.) or exp Shamanism/ or shaman*.mp. or traditional heal*.mp. or traditional food*.mp. or medicine man.mp. or medicine woman.mp. or autochtone*.mp. or (Native* adj1 (man or men or women or woman or boy* or girl* or adolescent* or youth or youths or person* or adult or people* or Indian* or Nation or tribe* or tribal or band or bands)).mp. **AND** exp Canada |
| **Web of Science** | Same as previous except “TS=” added prior to population and physical activity terms with “ALL=(Canada). |
| **Pubmed** | "First nations" or indigenous or aboriginal or "native peoples" or indian or eskimo or inuit or metis) or Indians, North American or Inuits or exp Health Services Indigenous or Ethnopharmacology or Athapaskan or Saulteaux or Wakashan or Cree or Dene or Inuit or Inuk or Inuvialuit* or Haida or Ktunaxa or Tsimshian or Gitsxan or Nisga'a or Haisla or Heiltsuk or Oweenkeno or Kwakwaka'wakw or Nuu chah nulth or Tsilhqot'in or Dakelh or Wet'suwet'en or Sekani or Dunne-za or Dene or Tahltan or Kaska or Tagish or Tutchone or Nuxalk or Salish or Stl'atlimc or Nlaka'pamux or Okanagan or Sec wepmc or Tlingit or Anishinaabe or Blackfoot or Nakoda or Tasttine or Tsuu T'inia or Gwich'in or Han or Tagish or Tutchone or Algonquin or Nipissing or Ojibwa or Potawatomi or Innu or Maliseet or Mi'kmaq or Micmac or Passamaquoddy or Haudenosaunee or Cayuga or Mohawk or Oneida or Onodaga or Seneca or Tuscarora or Wyandot or Aboriginal* or Indigenous* or Metis or red road or "on reserve" or off-reserve or First Nation or First Nations or Amerindian or or ethnomedicine or country food* or residential school* or traditional heal* Or traditional food* or medicine man or medicine woman or autochtone* **AND** (Activit* or Aerobic exercise or Anaerobic exercise or Aquatic exercise or Early Intervention or exercise or Exercise test or Exercise tolerance or Intervention study or Strength training or Cardiovascular training or Sports or Physical or Stretching exercise or Team sport or Treadmill exercise or fitness or strength training or cardiovascular training or lacrosse or wrestling or fishing or canoeing or archery or dance* or traditional games or hunt* or round dance or run* or jog* or leisure or recreation or "food gathering" or "physical activit*" or "hoop dancing") **AND** Canada |
| **OVID Embase** | (Activit* or Aerobic exercise or Anaerobic exercise or Aquatic exercise or Early Intervention or exercise or Exercise test or Exercise tolerance or Intervention study or Strength training or Cardiovascular training or Sports or Physical or Stretching exercise or Team sport or Treadmill exercise or fitness or strength training or cardiovascular training or lacrosse or wrestling or fishing or canoeing or archery or dance* or traditional games or hunt* or round dance or run* or jog* or leisure or recreation or "food gathering" or "physical activit*" or "hoop dancing").mp. **AND** ("First nations" or indigenous or aboriginal or "native peoples" or indian or eskimo or inuit or metis).mp. or exp Indians, North American/ or exp Inuits/ or exp Health Services, Indigenous/ or exp Ethnopharmacology/ or Athapaskan.mp. or Saulteaux.mp. or Wakashan.mp. or Cree.mp. or Dene.mp. or Inuit.mp. or Inuk.mp. or Inuvialuit*.mp. or Haida.mp. or Ktunaxa.mp. or Tsimshian.mp. or Gitsxan.mp. or Nisga'a.mp. or Haisla.mp. or Heiltsuk.mp. or Oweenkeno.mp. or Kwakwaka'wakw.mp. or Nuu chah nulth.mp. or Tsilhqot'in.mp. or Dakelh.mp. or Wet'suwet'en.mp. or Sekani.mp. or Dunne-za.mp. or Dene.mp. or Tahltan.mp. or Kaska.mp. or Tagish.mp. or Tutchone.mp. or Nuxalk.mp. or Salish.mp. or Stl'atlimc.mp. or Nlaka'pamux.mp. or Okanagan.mp. or Sec wepmc.mp. or Tlingit.mp. or Anishinaabe.mp. or Blackfoot.mp. or Nakoda.mp. or Tasttine.mp. or Tsuu T'inia.mp. or Gwich'in.mp. or Han.mp. or Tagish.mp. or Tutchone.mp. or Algonquin.mp. or Nipissing.mp. or Ojibwa.mp. or Potawatomi.mp. or Innu.mp. or Maliseet.mp. or Mi'kmaq.mp. or Micmac.mp. or Passamaquoddy.mp. or Haudenosaunee.mp. or Cayuga.mp. or Mohawk.mp. or Oneida.mp. or Onodaga.mp. or Seneca.mp. or Tuscarora.mp. or Wyandot.mp. or Aboriginal*.mp. or Indigenous*.mp. or Metis.mp. or red road.mp. or "on reserve".mp. or off-reserve.mp. or First Nation.mp. or First Nations.mp. or Amerindian.mp. or (urban adj3 (Indian* or Native* or Aboriginal*)).mp. or ethnomedicine.mp. or country food*.mp. or residential school*.mp. or ((exp Medicine, Traditional/ or traditional medicine*.mp.) not Chinese.mp.) or exp Shamanism/ or shaman*.mp. or traditional heal*.mp. or traditional food*.mp. or medicine man.mp. or medicine woman.mp. or autochtone*.mp. or (Native* adj1 (man or men or women or woman or boy* or girl* or adolescent* or youth or youths or person* or adult or people* or Indian* or Nation or tribe* or tribal or band or bands)).mp **AND** Canada.mp |
| **EBSCO Cinahl** | ( ( (MH "Eskimos") OR (MH "Native Americans") OR (MH "Indigenous Peoples+") or (MH "Health Services, Indigenous") or (MH "Indigenous Health") or (MH “Ethnopharmacology”) or Athapaskan or Saulteaux or Wakashan or Cree or Dene or Inuit or Inuk or Inuvialuit* or Haida or Ktunaxa or Tsimshian or Gitsxan or “Nisga'a” or Haisla or Heiltsuk or Oweenkeno or “Kwakwaka'wakw” or “Nuu chah nulth” or “Tsilhqot'in” or Dakelh or “Wet'suwet'en” or Sekani or “Dunne-za” or Dene or Tahltan or Kaska or Tagish or Tutchone or Nuxalk or Salish or “Stl'atlimc” or “Nlaka'pamux” or Okanagan or “Sec wepmc” or Tlingit or Anishinaabe or Blackfoot or Nakoda or Tasttine or “Tsuu T'inia” or “Gwich'in” or Han or Tagish or Tutchone or Algonquin or Nipissing or Ojibwa or Potawatomi or Innu or Maliseet or “Mi'kmaq” or Micmac or “Mic mac” or Passamaquoddy or Haudenosaunee or Cayuga or Mohawk or Oneida or Onodaga or Seneca or Tuscarora or Wyandot or Aboriginal* or Indigenous* or Metis or “red road” or "on reserve" or “off reserve” or “First Nation” or “First Nations” or Amerindian or (urban N3 (Indian* or Native* or Aboriginal*)) or ethnomedicine or “country food*” or “residential school*” or ((MH “Medicine, Traditional”) or "traditional medicine*" ) not Chinese ) or ( MH“Shamanism”) or shaman* or “traditional heal*” or “traditional food*” or “medicine man” or “medicine woman” or autochtone* or (Native* N1 (American* or man or men or women or woman or boy* or girl* or adolescent* or youth or youths or person* or adult or people* or Indian* or Nation or tribe* or tribal or band or bands)) ) ) AND ( ( (MH “Canada+”) or (Canad* or “British Columbia” or “Colombie Britannique” or Alberta or Saskatchewan or Manitoba or Ontario or Quebec or “Nova Scotia” or “New Brunswick” or Newfoundland or Labrador or “Prince Edward Island” or “Yukon Territory” or NWT or “Northwest Territories” or Nunavut or Nunavik or Nunatsiavut or NunatuKavut)) ) **AND** ((Activit* or Aerobic exercise or Anaerobic exercise or Aquatic exercise or Early Intervention or exercise or Exercise test or Exercise tolerance or Intervention study or Strength training or Cardiovascular training or Sports or Physical or Stretching exercise or Team sport or Treadmill exercise or fitness or strength training or cardiovascular training or lacrosse or wrestling or fishing or canoeing or archery or dance* or traditional games or hunt* or round dance or run* or jog* or leisure or recreation or "food gathering" or "physical activit*" or "hoop dancing")) **AND** Canada |

**References**

Campbell SM,  Dorgan M, and Tjosvold L. Filter to Retrieve Studies Related to Indigenous People of

Canada the OVID Medline Database. John W. Scott Health Sciences Library, University of Alberta. Rev. 05/05/2022.  <https://docs.google.com/document/d/1XqpWHN7hrFIyNwaqucRFRXaCnBOaeshFw4SR31Uxyek/edit>

Campbell, Sandy, Marlene Dorgan and Lisa Tjosvold. Filter to Retrieve Studies Related to Indigenous People of Canada the OVID EMBASE Database. John W. Scott Health Sciences Library, University of Alberta. Rev.10_04_2021. <https://docs.google.com/document/d/17CLxcrA90P8Q_7QpwblwqTAvqfmYaRRyMBFcuJGqPCc/edit>

Campbell, Sandy, Marlene Dorgan and Lisa Tjosvold. Filter to Retrieve Studies Related to Indigenous People of Canada the EBSCO CINAHL Database. John W. Scott Health Sciences Library, University of Alberta. Rev. October 10, 2021. [https://docs.google.com/document/d/17EZUiMOFKrhjn9XnOBZFjazCf8m7PCnKT0IeAp3Bkmg/edit#](https://docs.google.com/document/d/17EZUiMOFKrhjn9XnOBZFjazCf8m7PCnKT0IeAp3Bkmg/edit)
