# Supplementary material for: Physical activity–based, wholistic, wellness interventions for Indigenous women in Canada: An environmental scan to identify programs and promising practices
Source: Can J Public Health. 2025 Nov 6;117(4):610–33. doi: 10.17269/s41997-025-01091-9 (PMC13534398; doi:10.17269/s41997-025-01091-9)
Supplement: Supplementary file 2 [file 41997_2025_1091_MOESM2_ESM.docx]

**Supplementary Table B. Interview Responses**

| Program Questions | Physical activity-based programs for Indigenous women | | | | | | Programming not specific to Indigenous women | | | | | | | | | |
| --- | --- | --- | --- | --- | --- | --- | --- | --- | --- | --- | --- | --- | --- | --- | --- | --- |
| Province | **SK** | **ON** | **BC** | **PQ** | **AB** | **MB** | **AB** | **AB** | **PEI** | **ON** | **YUKON** | **NWT**** | **SK** | **NWT** | **NWT** | **PQ** |
| Description | Community-based health and wellness company in Saskatoon  Focus on functional fitness, sport programming, development and preparation for instructing and mentorship of trainers  3 Pillars: community, leadership, fitness  Target Indigenous youth and adult, but is open to everyone (note: mostly women attend) | Wholistic program, balance fitness: exercise and mindfulness – in tune to self  Mindfulness activities – meditation, stretches.  Nutrition component: talking about food, good foods, and bad foods - how to read label.  Uses "Healing food" book as a guide to discussed about emotional and physical and connection.  Recently changing program to train the trainer model - advocate wellness for people for the community.  No cycling in communities, once they are set, move on to training other community but does check ins | Walking program  Trauma-informed approach to PA  Social workers identify women.  Developed from feminist participatory research.  Front-line workers and community participatory approach | Kirano program: nutritionist would present program to community facilitator, implementation guide to support.  26wks  Concern about sustainability and is being reviewed.  Other issues: lack of publication and gaps in collecting feedback from facilitators | 15-week wholistic wellness program, with 52-week follow-up  Sessions once weekly plus pedometer use (walking) encouraged in between sessions.  Three components: PA (exposure to different types of fitness) - largest component, health education, sharing circles and ceremony.  Strong emphasis on capacity building  Facilitators paid community members | Walking Challenge: adult program, mostly women  Community sharing circle: focused on leadership, mental health, and connection.  Biking Programs: helps women gain independence and feel safer commuting.  Developing a land-based program for individuals under 30 years old (an expansion of what is currently offered in schools): equipment provided, participants learn to hunt, weave, trap, hunt, fish, etc.  Traditional Games Book: used by communities at festivals and also as a university textbook | Focus on mental health, healthy eating, physical activity.  Applying a health lens to Elementary and Jr. High Schools  Work with vulnerable communities identified by the school jurisdiction.  The school decides if the program would be a good fit and what their 'healthy school' vision looks like  The school is the driver of change, not the program itself, the school voices what they need.  Provide funds for staffing costs for 3 years, but staff are hired by the school.  Work closely with research - U of A - Paul J. Veugelers | Wholistic program: oral teachings and physical literacy through Indigenous games (have an Indigenous games kit) and drumming lessons.  Elders teach about traditional foods, crafts, making medicine bags, the medicine wheel, the seven sacred grandfather teachings (emotional/mental well-roundedness)  Adult programming: Dance and Drum and Calgary Young Offender Centre (CYOC)  Dance and Drum teaches respect and how to use the seven sacred teachings to find balance, create structure, and know their worth | Focus on youth.  Launching adult programming after gap identified, including:  1. Women's Wellness Day  2. Women's hike  3. Dads group (presently determining want- to date dads have wanted to do activities with their children as this is not as commonly role-modelled): First event is Axe Throwing (labeling them "Come Try" events)  4. Interest in swimming programs for moms  5. Lennox Islands Moms and Dads  6. Healthy cooking class with adults/parents  7. Micheal Thomas Walk and Run Club.  "Participants want to be part of something" | Focus on youth sport and wellness.  Recent focus on adults (more participation from women) after gap identified.  Boot camp at home, Weekly Wellness Warrior Challenges, virtual games  Master Indigenous Games  2018 North American Indigenous Games  Sports Pathways, Universities and Colleges  Recreational Programs from the Community  Programs for women and girls focusing on self-care and hygiene (mostly for youth, but want to engage adults in intellectual components such as book clubs)  Women and War Committee | Focus on youth and sports programming.  Some "open classes" that older adults can sign up for (not well attended)  Programming based on community demand.  Free, 5-session, adult yoga class, with an Indigenous instructor (mostly women attended) | Focus on coaching and athletics (competitive)  Youth and adult training courses delivered through the Coaching Association of Canada | Supports the development of community programming.  Not closely tied to communities as district acts as conduits  Leadership workshops for women | Focus on youth, but some events have an adult category (e.g., sports tournaments)  Women-specific programming available in all communities  Administrative and board development  Developing funding grants  Multisport camps: physical literacy, aquatic safety, ice safety  Physical Literacy Program: sponsored people to go international or Canadian conferences/summits to spread awareness and knowledge.  Rec Leader Training Camp  Community-based organization | Doesn’t provide direct services, only through training and support.  3 key areas:  1. “On the Land”: give autonomy, support, funding  2. “Professional Development”  3. “Active Communities”: Walk to XXX – Jan/Feb very hard to get people out and active; form teams of 10-20 people and conceptually walk the distance (1600ish km) of the big river (Mackenzie River); focus on 15min or more of moderate-vigorous cardiovascular activity  Health Fitness Leadership: goal was to certify people as Fitness Leaders based on Canadian Certification Program (currently being overhauled)  Focus on youth (e.g., canoe training/trips)  Intergenerational Programming Toolkit: having all ages involved increases motivation and enjoyment of activity.  Modules: Adapted from existing CCAA trainings ALEP, Falls and Balance, Functional Fitness and Long-term Care Facility (pilot project)  Elders in Motion: increase functional independence to combat isolation and increase community engagement.  Adapted to individual community needs.  Indigenous and Western knowledge on First Aid is incorporated.  Supporting Well-Being (in development): train on the land leaders to provide trauma-informed mental health support | Focus on family events, three types:  1."Wellness on the Land": explore outdoors, not specifically PA  2. Group family events: yoga, meditation, cooking  (group chats at the start led to storytelling and crafts)  3. Cooking brunch on Saturdays: post a recipe and then cook together  Preventative focus, modelled after Maori Navigator Concept  Prioritize families most in need |
| Aim | Build capacity in community.  Focus on consistency and functional fitness - pulling, lifting, moving, aerobic.  Improve metabolic conditions.  Training and certifying instructors  Creating a safe space for professional fitness to move mainstream | Increase wellness and build community | Increase PA  Expose women to new activities.  Provide social connection.  Provide community engagement.  Develop training program for service providers | Addressing obesity shame  Shift lens of nutrition from macro/micro count to just making better choices and eating when hungry  Change messaging to health rather than just losing weight | See mission and vision statement at www.wolftrail.ca.  Improve wellness and connection to community and culture for participants and program team | See mission and vision statement at [www.masrc.com/vision_and_mission](http://www.masrc.com/vision_and_mission)  Promoting sport sport and recreation as pillars to health and well-being  "Building partnerships, targeting community strategies, measuring success and creating community awareness" | "Create healthy kids in healthy schools" | "Physical literacy and making connection as family unit is a key priority" | Improve the wellness of community members based on idetified needs | N/A | Use funding to support sport and wellness activities for youth and adults | N/A | Support program development | Be the voice at territorial level for communities | Goal is to be champion of 'On the Land' programs | Mission is "to contribute to the holistic health, cultural strength and success of Indigenous families, individuals, and community living in the greater XXX area"  Engage and support families, illness prevention |
| Culture incorporation | Authenticity and intention in programming  Try to use the languages - Cree, Dene, Nakota | Sharing circle - checking in with participants, bringing healing  Open to all, but includes and promotes Indigenous culture, including Indigenous music | Trauma-informed, cultural grounding | N/A | Cultural components: Elder blessings and ceremony, sharing circles, Indigenous specific exercise classes when available  Guest speakers from Indigenous community | Sharing Circles  Land-based programming  Traditional Indigenous Games | Honouring Indigenous culture by using the circle of courage and Elder circles in the delivery of programming  Truth and Recognition Garden | Oral teachings  Elder teachings  Indigenous games  Smudge and Ceremonies for High School age population | Elders and ceremony involved in youth programming (e.g. smudging during girls' retreat)  No culture currently incorporated into adult programming.  Will celebrate history when ending the Micheal Thomas Walk and Run Club  Aboriginal Sports Council shares learnings (e.g., smudge, 7 Sacred Teachings - "The Elders always ground us by stating "every meeting today will affect 7 generations") | Reconciliation and cultural games for youth  Activating history and culture through games  Theme of reconciliation | Culture not intentionally incorporated | N/A | Dependent on community | Work with communities to incorporate cultural practices in welcome ceremony and throughout day.  Model ability to learn from others.  For cultural aspects, always ask don't assume | Majority of programming closely tied with culture, but sometimes there are people who are non-Indigenous that run programming in schools (canoe trip may not be cultural)  Collaborations to adapt their pre-existing program to be more culturally inclusive.  Opportunities must align with culture, or they will not work | Culture incorporated in many ways |
| Community involvement | Partner with a community coordinator Timely and effective communication helpful Important to partner with someone who is familiar with area and community Key partner is City of Saskatoon | Promotes local Indigenous culture.  Inclusive, bringing in other culture understanding, e.g., Ying-yang to connect with everyone during COVID-19 | Program development process: 1) environmental scan,2) formation of community advisory group. 3) core group of women that provides guidance throughout research processes and programming  Time on board is paid | Teams work under 'Health & Wellness' and less focus only on sports/athletics, this supports programming for communities | Community leads adapt program to their unique needs.  Facilitator come from community, with intimate knowledge of same  One Advisory Group or all programs (presently in 8 locations) - all come from Community.  Miskanawah primary community partner  Long-term strategy to move program oversight from partnership (University and Miskanawah) to fully in community | Using physical activity as a vehicle for social change  Community members run the program.  Sharing circles are open to the community | Community members, families, and school staff members are involved in initial meetings to determine the need/want for the program, focus on building connections with intention.  Offer resources and deliver programs in plain language/language that they understand.  Look at mental wellness rather than treating mental illness, identify strengths and build relationships, and provide resources to everyone including regional nurses and AHS staff.  Facilitators connect with community members who want to help out as ‘Champions’ (similar to Volunteers) who work alongside facilitators (who never run programs alone).  Offer Facilitators and Champions mental health/nutrition training and resources which they can share with the school community (including teachers). | Collaboration, mentorship, and leadership (in Indigenous and non-Indigenous cultural contexts)  Emphasis on teachings and family connection | Discuss needs with community members and partners, and develop programs accordingly | Indigenous Communities Active for Life Models: holistic coaching tool, introducing values from the medicine wheel | Community members able to suggest programming (such as the free yoga class) | Community development program for adults who want to get a program running.  Train facilitators in the North to host workshops (focus on delivering training course to adults who can then lead programming for youth) | Tribal councils | N/A | Facilitate a community meeting to discuss existing opportunities and gaps.  Create buy-in and a plan of action for the community | Community members suggest activities/programs |
| Enablers | Consistent programming important for attendance | "Having music is key to the program's success."  The wholistic nature is well received | Strong facilitator  Social workers identifying women and informing them.  People should be paid for facilitating and board work; peer leaders get honoraria for time, transportation, and childcare; advisory board positions seen as a job.  Consistent schedule (weekly at the same time)  Low commitment  Program facilitated additional visits for mothers with kids in foster care - lead could advocate to social worker, demonstrating that mother is connecting with other women and working on their health - women would meet baby and go on walk together. | Support from 'Health and Wellness'  Implementation manual | No cost  Childcare offered.  Facilitators well trained and nurturing community within their groups  Free step-counter and program water bottle and tee-shirt enjoyed | Free Fitbits, consistent use rewarded with free running shoes (25-27 pairs of shoes given out) | N/A | N/A | Offering prizes/incentives | N/A | N/A | N/A | Strong network supported programs, contributes to good uptake | N/A | NWT has good resources, and the government works well with local Indigenous governments. | N/A |
| Barriers | Ability of participant and community in general to meet threshold of Canadian food guide.  Cost to participants therefore partnerships with corporations or government to fund programming is essential.  Finding partners with the same vision who wants to support and own the program |  | Buy-in from senior management.  Vulnerable population already moves a lot,  "Fitness can be triggering, especially fitness classes" | Inadequate time to discuss | Transportation, although some support is provided it is a challenge for many.  Attaining long-term, non-grant dependent funding  Present program cycle found a major decrease in attendance when women who were coming in person had to convert to virtual | N/A | Evaluation is dependent on funding | Getting implementation across YMCAs throughout Canada | Transportation  Funding cycles  Facilities may be lacking in some places and good in others, facility costs can also vary | Internal struggles due to personnel turnover | N/A | N/A | N/A | Misconception that the recreation sector is a summer job/part-time, lots of departures in rec sector.  Rec personnel in communities take on broad job descriptions (low support but high demands) | NWT has a lot of turnover inherently as a territory  Interested in conducting more evaluations for grants, but face barriers such as participant IDs | N/A |
| Evaluation | Informal  Feedback from physical educators, community coordinator, participants - athletes, community members - inclusive of everyone involved | Informal  Through sharing circle, asking participants for feedback  Connect with communities and get feedback from trainers once per month | Self-reported PA levels  Sense of community scale  Semi-structured interviews and photovoice - "well received", "Healing is not linear" | Seeking to improve evaluation measures | Pre/post and 52-week evaluations  Mixed methods: photovoice (enjoyed greatly therefore will be used regardless of need to evaluate), anthropometric measures, fitness, comprehensive surveys at present.  Past studies report findings of increased confidence in exercising and improving fruits and vegetables (ref - Wicklum early paper) and program results in wellness and activation in a culturally safe environment (Wicklum NGT paper) | Measure success/metrics by whether it was enjoyable (participant numbers not important)  Ex. Biking program: train young people how to repair (learn new skills), track major destinations (where they took the bike) | Formal evaluation: Two-year check-in  Ongoing evaluation: Feedback from the school through surveys  Health action plans/goals are revised by the school a couple of times a year to see progress.  Long-term evaluation: depends on what is chosen to evaluate and expand on (to go deeper in the topic or wider and reach out to more communities) | Demographics  Record Mission and Action Stories  Do pre- and post-surveys, entered into FSI system with all programs (Ask: what do they want of this program and how did the program help?) | Demographics and attendance  No evaluations yet for adult programming  Use nationally established evaluations from the National Aboriginal Sport Circle, but there are none for adult programs | Fitness certification programming in SK  Evaluation done internally to report back to funders | N/A - pilot program, in the future will measure social impact | N/A | N/A | Pre-, post-, and during  Daily in-person check-ins and check-ins at the end of summer  Trying to expand evaluation on programs, evaluate host groups (support, needs, what went well/didn’t go well) | Collaborative has a reporting template  Trainings do verbal feedback  Supporting Well-being in process of having an evaluation created (2023)  Occasionally will conduct an evaluation of grant program when required  Do not conduct “proper” research evaluation  Conduct a lot of surveys and chats with community members  Constantly “evaluating” and tweaking programming to be the most community-based possible.  Program leads determine (based on funding) if they run an evaluation | Conversation-based/organically (not through forms or surveys)  Inspired by Wabano (Ottawa)- participants given 1 stone and there are 3 jars representing satisfaction/emotion and they drop their stone into the corresponding one |
| Marketing methods | Word of mouth most significant  Website | Website  Reaching audiences through friendship contracts, Word of mouth  Marketing is a process; you must be willing to go into the community and introduce yourself and build relationships | Word of mouth  Posters  Currently in-progress of creating small documentary; animated video on trauma. - did this get done??, try to find | N/A | Word of mouth  Facebook  Posters  Email distribution to relevant agencies and organisations that can identify women for the program | Facebook and Instagram  Thrive on “unconventional” means of contact- If they have a contact with a dependable line, will use that connection platform (anything goes!)  Newsletter (internally and externally to communities and prov/fed government) | Program website  School jurisdictions identify vulnerable schools.  If the school is interested, meetings and a presentation are hosted where parents, community members, and staff members can learn about the program | Word of mouth  Website  Posters | Word of mouth  Facebook  Website  Community leaders/champions  "Need to know what your community uses" | Facebook (most popular for remote communities)  Connections within communities  Word of mouth  Regional Coordinators  Friendship Centres | Facebook, Instagram  Posters  Word of mouth  Website (new- not relied on) | N/A | N/A | Facebook (most effective)  Newspaper (for jobs)  Radio (for bigger events) | Facebook  RPA social media  Website (blog posts)  Walk to XXX uses many promotional tools (Facebook, Instagram, Radio, newsletter) | Word of mouth  Facebook  Email listserv |
| How did you decide which programs to run? | Based on community need | "Have a menu of programs that community can chose from..."  "Need to know about community - networking, raising awareness based on what the community needs are." | Consulting with women in Advisory group and board, created appropriate program | N/A | Need identified in original community | N/A | The community gets a say even before the school decides to run the program.  Schools have the autonomy to decide what to run | Drum and Dance program: had over 25 emails from people asking for it  CYOC program: one of the guards asked because the kids were asking.  "Making and having a connection with people is the incentive for them to want to keep coming back" | Based on participant and community requests | N/A | Input from community | N/A | Communities decided | N/A | N/A | N/A |
| Funding | For profit program but funded through other organisations so participants do not have to pay | Local community sponsor, but free or low cost for participants or low cost to participants  Has private pay clients also  Barrier: fee for participants to rent equipment to take home | SSHRC grant | Initial funding through government Not funded presently.  Staff will go to communities to help identify facilitators and implement program | Provincial and federal grants and private and corporate donors | Provincial and federal funding, also individual grants (not typically); large backer is Lotteries of Manitoba | Grants from donors  Oil and Gas company working in the community.  Federal government (PHAC) | YMCA is donation and grant-funded (no separate fundraising)  Collaborate with all YMCA organizations for campaigns and to give back | Provincial and Federal Government | All provinces/territories are funded differently.  Mainly public funding that changes annually  A larger amount comes from the Province and Ministry of Tourism, Culture and Sport  Industry (Unilever, Can Tire, Giant Tiger) | Sports Canada  Sport and Social Development in Indigenous Communities (SSDIC) | N/A | Sask Lotteries Trust Fund  Disseminates funds to districts (7 in the province) | Lotteries  Government  Grants  Will charge a participant fee for signature events | Non-profit wit lots of sourced  Core funding is from lotteries and federal and territorial government.  Grants  Funders across the NWT (non-profits/NGOs)  ARC (Active Resilient Connected during COVID) - communities apply for small grants to run small PA-oriented events | Not-for-profit with multiple funders |
| Strategic partnerships, challenges and enablers | City of Saskatoon  Saskatchewan Parks and Recreation Association | Individual connections  Partner with community organisations who will sponsor.  No large corporation sponsorships sought |  | N/A | Local Indigenous community organisations e.g., Miskanwah  Indigenous health organisations  Presently PHAC | N/A | Knowledge exchange event.  When reaching out to Oil and Gas sector, looked at companies whose vision aligned with the program and would invest in their goals | Partnerships with Elders and instructors  Only YMCA in Canada to have this department for 12 years | Local communities/First Nations  Lennox Island and Abeegweit First Nation  Native Council of PEI  Provincial sports organisations (e.g. Lacrosse PEI, Aboriginal Sports Council) | Utilized during COVID (Bauer activity program, Canadian Tire)  IFCs are more unique to regional coordinators | Sports Canada  Local communities/First Nations  Whitehorse recreation department  Territorial sports organisations  Volunteer organisations  Schools | N/A | Tribal councils | Schools  Friendship centres  Indigenous governance  Rec centres  Territorial organizations  Band managers  SAOs (Senior administrative officers) | Make Way  Social Services  Environmental and Natural Resources  Nations  Regional corporations  “Hotıì ts’eeda is a research support unit hosted by the Tłı̨chǫ Government and governed primarily by Northwest Territories (NWT) Indigenous Governments. Hotıì ts’eeda connects NWT organizations, and communities with researchers and funding to achieve health research and training goals. It is funded by the Canadian Institutes of Health Research.”  Aurora College  Dene Nation  NWT Seniors Society  Literacy Council  Canadian Centre for Activity and Aging  AFLCA  Aboriginal Sport Circle  Mackenzie Rec Association  Governments of all kinds | Important for partners to "truly have the interests of the program in mind."  Women's clinic  Local College  Local farm |
| COVID-19 changes | Program on hold, gradual re-entry | Virtual delivery | Program on hold | N/A | Program converted to virtual, but attendance declined significantly | N/A | During COVID-19, sent surveys to schools asking if they need support (Mental health resources, how to promote health at home with family, etc.) | Went virtual (Zoom) | Started the learn to run the program online | Unable to run many activities.  Did not seem to shift online | N/A (yoga was pre-covid19 pandemic) | N/A | N/A | Moved online.  Some communities have internet issues- used “MiFi” a Bell internet services that improves Wi-Fi services | Get Active Program can borrow equipment for online events or host socially distanced activities | Some activities stopped; some went online |

** interview interrupted and unable to reschedule
